# A spinal neural circuitry for converting touch to itch sensation

**DOI:** 10.1038/s41467-020-18895-7

**Published:** 2020-10-08

**Authors:** Sihan Chen, Xiao-Fei Gao, Yuxi Zhou, Ben-Long Liu, Xian-Yu Liu, Yufen Zhang, Devin M. Barry, Kun Liu, Yingfu Jiao, Rita Bardoni, Weifeng Yu, Zhou-Feng Chen

**Affiliations:** 1 Center for the Study of Itch and Sensory Disorders, Washington University School of Medicine, St. Louis, MO 63110 USA; 2 Department of Anesthesiology, Washington University School of Medicine, St. Louis, MO 63110 USA; 3 Department of Anesthesiology, Renji Hospital, School of Medicine, Shanghai Jiaotong University, Shanghai, 200127 China; 4 Translational Research Institute of Brain and Brain-Like Intelligence, Department of Anesthesiology, Shanghai Fourth People’s Hospital Affiliated to Tongji University School of Medicine, 200434 Shanghai, China; 5 Department of Neurobiology, School of Basic Medicine and Tongji Medical College, Huazhong University of Science and Technology, Wuhan, P. R. China; 6 Department of Biomedical, Metabolic and Neural Sciences, University of Modena and Reggio Emilia, 41125 Modena, Italy; 7 Department of Psychiatry, Washington University School of Medicine, St. Louis, MO 63110 USA; 8 Department of Developmental Biology, Washington University School of Medicine, St. Louis, MO 63110 USA

**Keywords:** Molecular biology, Neuroscience, Sensory processing

## Abstract

Touch and itch sensations are crucial for evoking defensive and emotional responses, and light tactile touch may induce unpleasant itch sensations (mechanical itch or alloknesis). The neural substrate for touch-to-itch conversion in the spinal cord remains elusive. We report that spinal interneurons expressing *Tachykinin 2-Cre* (*Tac2*^*Cre*^) receive direct Aβ low threshold mechanoreceptor (LTMR) input and form monosynaptic connections with GRPR neurons. Ablation or inhibition markedly reduces mechanical but not acute chemical itch nor noxious touch information. Chemogenetic inhibition of *Tac2*^*Cre*^ neurons also displays pronounced deficit in chronic dry skin itch, a type of chemical itch in mice. Consistently, ablation of gastrin-releasing peptide receptor (GRPR) neurons, which are essential for transmitting chemical itch, also abolishes mechanical itch. Together, these results suggest that innocuous touch and chemical itch information converge on GRPR neurons and thus map an exquisite spinal circuitry hard-wired for converting innocuous touch to irritating itch.

## Abstract (web summary)

Light touch can sometimes induce unpleasant itch sensation but the neural correlates of this conversion are not well studied. Here, the authors provide evidence that *Tac2* expressing spinal interneurons are connected to gastrin-releasing peptide receptor (GRPR) neurons and form an integral part of the neural circuit underlying touch evoked itch.

## Introduction

Itch invariably provokes the urge to scratch, while bodily responses to touch are remarkably diverse and influenced by multifaceted factors, such as the nature and intensity of tactile stimuli as well as the areas stimulated. Both touch and itch can elicit a defensive response, and are important for animal survival and welfare^1–4^. While itch and touch are encoded through distinct neuronal pathways from the periphery to the brain^5–9^, one fascinating observation is that a nonitchy stimulus such as a light touch may evoke an unpleasant itch sensation on the hairy skin^10–12^, known as mechanical itch or alloknesis. Mechanical itch presumably informs animals of external environmental irritants (e.g., insects and wool)^13,14^. Physical contact with textile fibers such as wool or cotton swabs from the seemingly normal skin area neighboring the itchy skin often evokes itching sensation^15^. Because alloknesis could be intolerable under pathological itch conditions, it could be a presenting problem for patients with chronic itch, including atopic dermatitis, dry skin itch, and urticaria^3,16–20^. One plausible mechanism is the excitation of the spinal interneurons, which receive input from low threshold mechanoreceptors (LTMRs)^11,13,21^.

Gastrin releasing peptide (GRP) is an itch-specific peptide in sensory neurons and can activate its receptor GRPR in the spinal cord to relay nonhistaminergic itch information to the brain^5,9,22^, whereas neuromedin B (NMB) and its receptor (NMBR) mediate histamine-evoked itch via GRPR neurons^23,24^. Loss-of-function and gain-of-function studies demonstrate that the GRP–GRPR neuronal pathway is an itch-specific pathway responsible for both histaminergic and nonhistaminergic itch transmission^5,6^. Recent studies have shown that *Ucn3*^*tdTom*^ lineage neurons in the spinal cord are essential to transmitting mechanical itch via Toll-like receptor 5 (TLR5) Aβ-LTMRs independent of GRPR neurons^25,26^. The spinal neurons expressing neuropeptide Y (NPY) and its receptor NPY receptor 1 (NPY1R) have been shown to be important for gating mechanical itch^21,27^. On the other hand, recent studies have also implicated the NPY–NPY1R signaling in inhibition of chemical itch^28,29^, raising the possibility that mechanical itch is converged on GRPR neurons. Piezo-2, a mechanotransduction ion channel in Merkel cell complex^30^, acts as an inhibitory channel for gating touch-to-itch conversion, as well as aging-associated alloknesis^31^. Because *Ucn3*^*tdTom*^ neurons are developmental lineage neurons and widespread in the dorsal horn, the question of whether there exists a subpopulation of neurons for touch-to-itch conversion which can be unequivocally identified in a lamina-specific pattern remains to be determined. Furthermore, conflicting results concerning the function of NPY in gating chemical itch prompted us to reappraise the role of GRPR neurons in mechanical itch.

The spinal cord interneurons expressing *Tachykinin 2* (*Tac2*), which encodes neuropeptide neurokinin B (NKB)^32^, are exclusively located in the LTMR recipient-zone (RZ)^7,33^. In this study, we aimed to test the hypothesis that *Tac2* neurons are required for mediating mechanical itch. Our study reveals that *Tac2* neurons receive Aβ LTMR inputs and are required for mechanical, but not chemical, itch under normal physiological condition. However, *Tac2* neurons located in lamina IIi are activated to participate in chemical and mechanical itch only under pathological itch conditions. Contrary to previous studies, we find that GRPR neurons are essential for mediating mechanical itch and function downstream of *Tac2* neurons to convert innocuous touch to irritating itch.

## Results

### *Tac2* neurons are activated by mechanical itch stimulation

Spinal cord dorsal horn can be divided into distinct laminae according to molecular expression profile, afferent projection and functional allotment^34^. *Tac2* neurons are a subset of interneurons that forms a distinct band encompassing the inner layer of lamina II (IIi) and the outer layer of lamina III (IIIo) in the spinal cord (Supplementary Fig. 1a, b)^33,35^. Consistent with the previous study^33^, immunohistochemistry (IHC) shows the overwhelming majority (283/349, 81.1%) of the spinal *Tac2* neurons of mice derived from the mating of *Tac2*^*Cre*^ mice with Ai9 reporter mice^36^ (hereafter referred to as *Tac2*^*tdTom*^ neurons) express Lmx1b, a transcription factor expressed in glutamatergic interneurons in the spinal cord and brainstem (Supplementary Fig. 1a)^37–39^, while very few (5/386, 1.3%) express Pax2, an inhibitory neuronal marker (Supplementary Fig. 1b)^39^. RNA scope in situ hybridization (ISH) showed that almost all *Tac2*^+^ neurons (288/295, 97.6%) in the superficial dorsal spinal horn express *Vglut2*, a marker for excitatory neurons (Supplementary Fig. 1c). This is consistent with RNAseq result^40^, 60.7% (269/443) of which also express *Vgat* (Supplementary Fig. 1d).

**Fig. 1:**
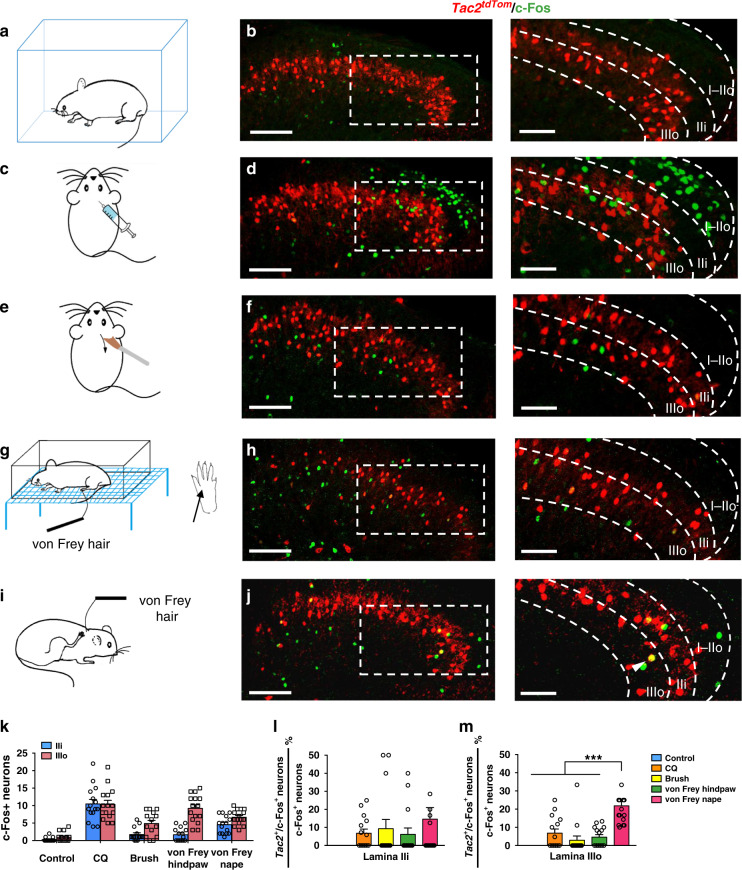
*Tac2*^*Cre*^ neurons in the spinal cord are activated by mechanical itch stimulation. **a**, **c**, **e**, **g**, **i** Schematic of mice in free ambulating state (**a**), i.d. CQ injection (**c**), soft brushing (**e**), von Frey hair applied to the hindpaw (**g**), von Frey hair applied to the hairy skin (**i**). **b**, **d**, **f**, **h**, **j** Representative images of c-Fos expression (green) in the spinal cord of *Tac2*^*tdTom*^ (red) mice corresponding to **a**, **c**, **e**, **g**, **i**, respectively. Right: higher magnification of the boxed area. Scale bars, 100 μm (left); 50 μm (right). *n* = 3 mice per group. **k** Comparison of the total number of c-Fos positive neurons in laminae IIi and IIIo under different conditions. *n* = 15 sections from three mice per group. **l**, **m** Comparison of the percentage of *Tac2*^*tdTom*^ and c-Fos double positive neurons in c-Fos positive neurons in lamina IIi (**l**) and IIIo (**m**) under different conditions. *n* = 15 sections from three mice per group. one-way ANOVA with Tukey *post-hoc*, ***p = 0.0001. All data are presented as mean ± s.e.m. and error bars represent s.e.m. Source data are provided as a Source Data file.

To test which sensory modality transmission may require *Tac2* neurons, we used c-Fos, a neuronal activity marker, as a surrogate to determine whether *Tac2*^*tdTom*^ neurons are activated in response to different types of stimuli. As a control, a free ambulating mouse without evoked stimuli showed little c-Fos activity, as detected by IHC, in the superficial dorsal horn (Fig. 1a, b and Supplementary Fig. 2a). Intradermal injection (i.d.) of chloroquine (CQ), an archetypal pruritogen for chemical itch, evoked robust c-Fos activity, mostly restricted to laminae I and IIo (Fig. 1c, d and Supplementary Fig. 2b). However, c-Fos was barely detected in *Tac2*^*tdTom*^ neurons (lamina IIi: 6.7 ± 2.9%; lamina IIIo: 6.5 ± 3.0%) (Fig. 1l, m), consistent with an earlier report that *Tac2* is not required for chemical itch^33^. Next, we examined c-Fos induced by mechanical dynamic stimulus using brushing at 2 cm s^−1^ (Fig. 1e)^41^. While most c-Fos^+^ neurons were found in laminae IIi–IIIo (Fig. 1f and Supplementary Fig. 2c), few were located in *Tac2*^*tdTom*^ neurons (lamina IIi: 9.4 ± 4.7%; lamina IIIo: 3.2 ± 3.0%)(Fig. 1l, m). To determine whether *Tac2*^*tdTom*^ neurons are involved in detecting and transmitting noxious mechanical information, the hindpaw of the mouse was poked with a von Frey filament (1.4 g) (Fig. 1g). Although a von Frey filament evoked c-Fos expression across laminae I–III (Fig. 1h and Supplementary Fig. 2d), there was little co-expression with *Tac2*^*tdTom*^ neurons (lamina IIi: 6.7 ± 3.7%; lamina IIIo: 5.0 ± 2.3%) (Fig. 1l, m). Lastly, we tested whether *Tac2*^*tdTom*^ neurons are required for mechanical itch evoked by applying a von Frey filament (0.07 g) to the hairy skin of the nape (Fig. 1i)^42^. Mechanical itch induced c-Fos expression across laminae I-III (Fig. 1j and Supplementary Fig. 2e). Notably, compared to other stimuli tested, significant amounts of c-Fos were found in *Tac2*^*tdTom*^ neurons (laminae IIi: 14.6 ± 6.4%; IIIo: 21.8 ± 3.7%) (Fig. 1l, m). Interestingly, comparison of c-Fos activity in response to chemical itch and innocuous or noxious touch stimuli suggests that overall *Tac2*^*tdTom*^ neurons in lamina IIIo are more active than IIi in response to touch stimuli (Fig. 1k), and *Tac2*^*tdTom*^ neurons are prone to be activated by mechanical itch-related touch stimulation (Fig. 1l, m). Taken together, these results indicate that *Tac2*^*tdTom*^ neurons are more likely to be involved in mechanical itch transmission.

### Electrophysiological properties of *Tac2* neurons

**Fig. 2:**
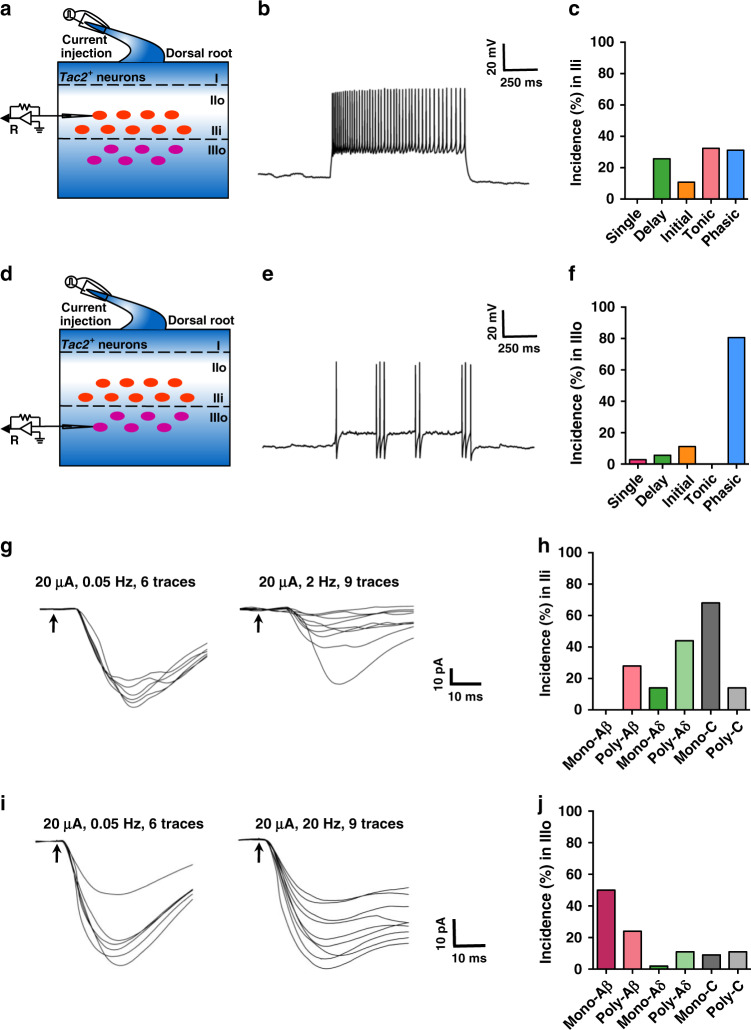
Electrophysiological firing patterns and fiber inputs of *Tac2*^*tdTom*^ neurons. **a**, **d** Schematic of the patch-clamp recording of *Tac2*^*tdTom*^ neurons selected from lamina IIi (**a**) and lamina IIIo (**d**). **b**, **e** Representative trace of tonic firing pattern (**b**) and phasic-bursting firing pattern (**e**) at 40 pA. Rheobase was 20 pA. **c** Percentages of different type of firing patterns of *Tac2*^*tdTom*^ neurons in lamina IIi. *n* = 74 neurons. **f** Percentages of different type of firing patterns of *Tac2*^*tdTom*^ neurons in lamina IIIo. *n* = 36 neurons. **g** Representative traces showing one *Tac2*^*tdTom*^ neuron in lamina IIi receiving poly-low threshold-Aδ input. It could not follow 20 μA/2 Hz stimulation. The latency was between 6 and 10 ms. **h** Percentage of each type of input onto *Tac2*^*tdTom*^ neurons in lamina IIi. *n* = 50 neurons from 5 mice. **i** Representative traces showing one *Tac2*^*tdTom*^ neuron in lamina IIIo receiving mono-Aβ input. It followed 20 μA/20 Hz stimulation. The latency was less than 6 ms. **j** Percentage of each types of input onto *Tac2*^*tdTom*^ neurons in lamina IIIo. *n* = 46 neurons from 5 mice. Source data are provided as a Source Data file.

Next, we examined electrophysiological properties of *Tac2*^*tdTom*^ neurons using whole-cell patch-clamp recording^23^. *Tac2*^*tdTom*^ neurons form a cell band between lamina IIi and IIIo (Supplementary Fig. 1a, b). To avoid mingled lamina II and III *Tac2*^*tdTom*^ neurons, we recorded the neurons at the edges of cell band. The neurons at top edge (in translucent band) are considered as lamina IIi *Tac2*^*tdTom*^ neurons, while those at bottom edge of *Tac2*^*tdTom*^ neurons band are considered as lamina IIIo neurons. This enabled us to identify and record *Tac2*^*tdTom*^ neurons in these two regions discretely (Fig. 2a, d). *Tac2*^*tdTom*^ neurons in lamina IIi displayed four distinct firing patterns: delayed firing (25.7%, 19 of 74), initial firing (10.8%, 8 of 74), tonic firing (31.1%, 23 of 74) and phasic-bursting pattern (24.0%, 19 of 74) (Fig. 2b, c). More strikingly, the dominating firing pattern of *Tac2*^*tdTom*^ neurons in lamina IIIo is phasic-bursting (80.6%, 29 of 36) (Fig. 2e, f), whereas the rest comprising single spiking (2.8%, 1/36), delayed firing (5.6%, 2 of 36), and initial bursting (11.1%, 4 of 36) (Fig. 2f). The observation that a vast majority of lamina IIIo *Tac2* neurons show the same firing pattern is highly unusual, given the heterogeneity of the firing patterns for lamina III interneurons as shown previously^7,21^.

The location of *Tac2*^*tdTom*^ neurons suggests that they may receive mono-LTMR Aβ input. To evaluate the nature of LTMR Aβ primary afferent inputs for *Tac2*^*tdTom*^ neurons, we adopted a root stimulation protocol that allowed the best preservation of Aβ fibers^43^ and recorded *Tac2*^*tdTom*^ neurons from a thick parasagittal spinal cord slice (550 µm) with the root attached. Interestingly, *Tac2*^*tdTom*^ in lamina IIi predominantly receive monosynaptic C fiber input (68.0%, 34 of 50), followed by polysynaptic Aδ inputs (44.0%, 22 of 50) and polysynaptic Aβ inputs (28.0%, 14 of 50) (Fig. 2g, h). However, the majority of *Tac2*^*tdTom*^ neurons in lamina IIIo, received either monosynaptic (50.0%, 23 of 46) or polysynaptic Aβ inputs (23.9%, 11 of 46) (Fig. 2i, j), while the rest received negligible Aδ and C fiber inputs (Fig. 2j). These results revealed different properties of *Tac2*^*tdTom*^ in lamina IIIo and IIi and suggest that *Tac2*^*tdTom*^ in lamina IIIo represent a population of excitatory interneurons that are ideally suitable for conveying and processing the intensity and the duration of Aβ afferent excitation.

### Opto-activation of *Tac2* neurons evokes scratching behaviors

**Fig. 3:**
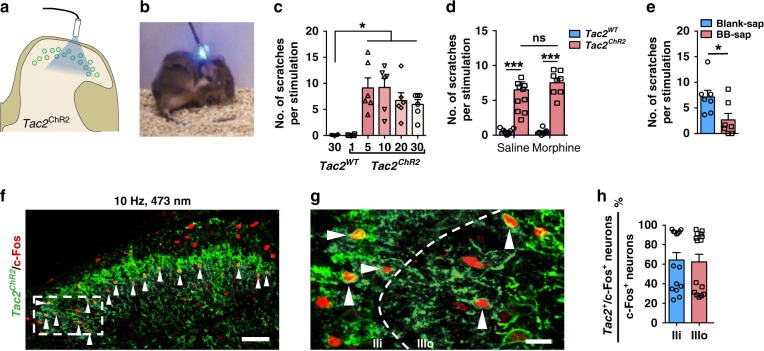
Optostimulation of *Tac2* neurons evoked itch-related scratching behavior. **a** Schematic of blue light stimulation of *Tac2*^*ChR2*^ neurons. **b**, **c** A snapshot (**b**) and quantification (**c**) of blue light-induced scratching behaviors in *Tac2*^*ChR2*^ mice. One-way ANOVA with Tukey *post*-*hoc*, *p = 0.012. *n* = 6 mice per group. **d** The effect of i.t. morphine on blue light-induced scratches. Two-way ANOVA with Bonferroni *post-hoc*, ***p = 0.00001, *n* = 9 for *Tac2*^*WT*^/saline, *n* = 7 for *Tac2*^*WT*^/morphine, *n* = 11 for *Tac2*^*ChR2*^/saline, *n* = 8 for *Tac2*^*ChR2*^/morphine. **e** Blue light-induced scratches decreased after BB-sap treatment. Two-tailed Student’s unpaired *t-*test, *p = 0.029, *n* = 7 mice per group. **f** Double IHC of c-Fos (red) and GFP (green) in the spinal cord of *Tac2*^*ChR2*^ mice following blue light stimulation. **g** Higher magnification of the boxed area in **f**. Arrowheads indicate double-stained c-Fos^+^/*Tac2*^*ChR2+*^ neurons in **f**, **g**. Scale bars, 50 μm in **f** and 10 μm in **g**. **h** Quantification of percentages of *Tac2*^*ChR2*^/c-Fos double positive neurons in c-Fos positive neurons in laminae IIi and IIIo, respectively. *n* = 3 mice. All data are presented as means ± s.e.m. and error bars represent s.e.m. Source data are provided as a Source Data file.

To determine the role of *Tac2* neurons in itch, we examined behavioral response of mice derived from mating between *Tac2*^*Cre*^ mice and Ai32 reporter mice expressing channel rhodopsin-eYFP^44^ (ChR2-eYFP, referred to as *Tac2*^*ChR2*^) using optogenetic approach (Fig. 3a, b). Optoactivation of *Tac2*^*ChR2*^ neurons provoked scratching bouts, starting at 5 Hz and increasing until 10 Hz (Fig. 3c), revealing that the capacity of *Tac2*^*ChR2*^ neurons to induce scratching bouts reaches a limit at 10 Hz. To determine whether evoked scratching behavior reflects pain or itch, intrathecal (i.t.) injection of morphine was performed to inhibit the spinal nociceptive transmission. I.t. morphine failed to attenuate evoked scratching behaviors at 5 Hz (Fig. 3d), indicating that scratching behavior is likely to be related to itch rather than pain. We also examined whether evoked scratching behavior is itch-related by ablating spinal GRPR neurons with i.t. bombesin-saporin (BB-sap)^6^. Indeed, the scratching behavior induced by optostimulation of *Tac2*^*ChR2*^ neurons was significantly attenuated (Fig. 3e), suggesting that the evoked scratching behavior was at least partially dependent on GRPR neurons. To confirm whether *Tac2*^*ChR2*^ neurons were indeed activated by optostimulation, we examined the expression of c-Fos using IHC following blue light stimulation. Notably, c-Fos was observed across the dorsal horn laminae, reminiscent of c-Fos pattern induced by mechanical itch (Fig. 1j, 3f, g). Remarkably, most c-Fos positive neurons in laminae IIi-IIIo were *Tac2*^*ChR2*^ neurons (Fig. 3f–h). Given only a few scratches evoked per ten stimulation in contrast to robust scratching bouts induced by chemical itch, these findings suggest that activation of *Tac2* neurons could mimic von Frey-evoked scratching.

### Inhibition of *Tac2*^*Cre*^ neurons attenuates mechanical itch

**Fig. 4:**
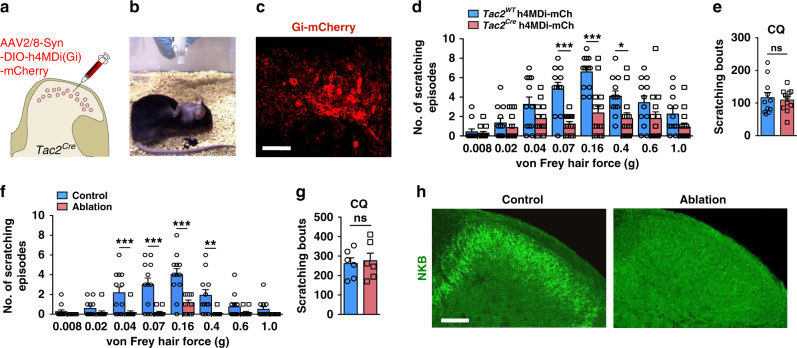
Inhibition of *Tac2* neurons in the spinal cord attenuated mechanical itch. **a** Schematic of intraspinal injection. **b** A snapshot of mouse with scratching behavior induced by von Frey filament. **c** IHC image of mCherry^+^ neurons (red) in the cervical spinal cord of *Tac2*^*Cre*^ mice injected with hM4Di-mCherry virus. All 14 mice with virus injections were subjected to IHC confirmation. Scale bar, 50 μm. **d**, **e** Mechanical itch test (**d**) and CQ itch test (**e**) after chemogenetic inhibition of *Tac2*^*Cre*^ neurons. (**d** two-way ANOVA with Bonferroni *post*-*hoc*, *p = 0.019, ***p = 0.0001, *n* = 12 mice for *Tac2*^*WT*^ and *n* = 14 mice for *Tac2*^*Cre*^; **e** unpaired two-tailed Student’s *t-*test, *p* = 0.72, *n* = 11 mice per group, ns not significant). **f**, **g** Mechanical itch test (**f**) and CQ itch test (**g**) after conditional ablation of *Tac2*^*Cre*^ neurons in the spinal cord. (**f** two-way ANOVA with Bonferroni *post*-*hoc*, **p = 0.002, ***p = 0.0001, *n* = 12 mice per group; **g** unpaired two-tailed Student’s *t-*test, *p* = 0.78, *n* = 6 mice per group, ns not significant). **h** IHC of NKB (green) in the cervical spinal cord of control mice (left) and mice with conditional ablation of *Tac2*^*Cre*^ neurons (right). Scale bar, 100 μm. All data are presented as means ± s.e.m. and error bars represent s.e.m. Source data are provided as a Source Data file.

Next we used Cre-dependent G_i_-coupled designer receptors exclusively activated by designer drugs (DREADDs)^45^ to inhibit *Tac2*^*Cre*^ neurons followed by intraspinal injection of adeno-associated viruses (AAV2/8-Syn-DIO-h4MDi (Gi)-mCherry) into the cervical cord of mice (Fig. 4a–c). While the baseline of mechanical itch and CQ itch remained the same after virus injection, clozapine injection significantly reduced mechanical itch elicited by von Frey hair stimulation (from 0.07 to 0.4 g) (Fig. 4d and Supplementary movie 1). In contrast, CQ-induced itch was not affected (Fig. 4e).

To test whether inhibition of *Tac2*^*Cre*^ neurons would alter mechanical thresholds and thermal pain, AAV2/8-Syn-DIO-h4MDi (Gi)-mCherry virus was injected into the lumbar spinal cord of *Tac2*^*Cre*^ mice to selectively inhibit lumbar *Tac2*^*Cre*^ neurons (Supplementary Fig. 3g). No significant difference was detected in mechanical thresholds (Supplementary Fig. 3h) or thermal pain (Supplementary Fig. 3i) between the saline-treated and clozapine-treated groups, indicating that *Tac2*^*Cre*^ neurons may be selectively required for mechanical itch transmission.

Lastly, intersectional genetic approach was employed to ablate *Tac2*^*Cre*^ neurons in the spinal cord of *Tac2*^*Cre*^ mice^46^. Strikingly, mechanical itch evoked by von Frey hair force was almost abolished after intraperitoneal (i.p.) injection of diphtheria toxin (DTX) in the *Lbx1-Flpo/Tau-DTR/Tac2-Cre* mice (Fig. 4f)^41^. In contrast, ablation of *Tac2*^*Cre*^ neurons did not change CQ-induced itch (Fig. 4g), nor pain or motor behaviors (Supplementary Fig. 3a–f), consistent with previous studies^33^. The complete ablation of *Tac2*^*Cre*^ neurons was confirmed by the absence of NKB immunostaining (Fig. 4h).

### Mechanical itch is dependent on GRPR neurons

**Fig. 5:**
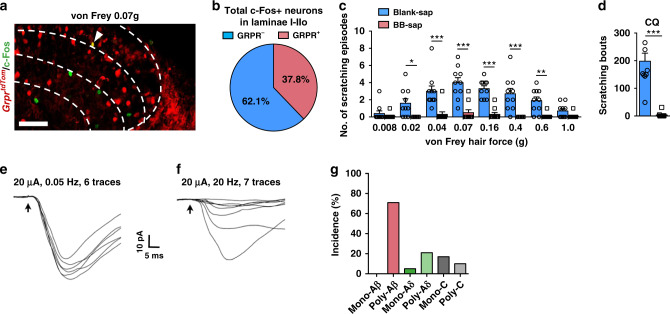
Mechanical itch depends on GRPR neurons. **a** Representative image of c-Fos IHC (green) in the dorsal horn of *Grpr*^*tdTom*^ (red) mice after von Frey hair stimulation (0.07 g) on the nape skin. Arrowhead indicates c-Fos^+^/ *Grpr*^*tdTom* +^ neuron. *n* = 3 mice. Scale bar, 50 μm. **b** Quantification of the percentage of *Grpr*^*tdTom* +^ /c-Fos^+^ neurons in c-Fos^+^ neurons in laminae I–IIo. *n* = 3 mice. **c**, **d** Mechanical itch test (**c**) and CQ itch test (**d**) after 500 ng of BB-sap treatment. (**c** two-way ANOVA with Bonferroni *post*-*hoc*, *p = 0.018, **p = 0.005, ***p = 0.00001, *n* = 10 mice per group; **d** unpaired two-tailed Student’s *t-*test, ***p = 0.00001, *n* = 10 mice per group). **e**, **f** Representative traces showing one *Grpr*^*tdTom*^ neuron cannot follow 20 µA/20 Hz stimulation. **g** Percentage of each types of inputs onto *Grpr*^*tdTom*^ neurons. Data from five mice, *n* = 42 neurons. All data are presented as means ± s.e.m. and error bars represent s.e.m. Source data are provided as a Source Data file.

While mechanical itch and chemical itch have been considered to function through distinct neuronal pathways in the spinal cord^26,27,46^, recent studies showed that NPY–NPY1R signaling can inhibit both mechanical and chemical itch, indicating that they may share the same pathway^28,29^. The finding that ablation of GRPR neurons significantly reduced the scratching behavior evoked by optoactivation of *Tac2*^*ChR2*^ neurons prompted us to examine whether mechanical itch transmission is dependent on GRPR neurons. We first examined whether c-Fos is activated in GRPR neurons using *Grpr*^*tdTom*^ mice^47^. Von Frey hair stimulation applied to the hairy skin of the nape induced c-Fos expression in laminae I–II, including *Grpr*^*tdTom*^ neurons (Fig. 5a), Throughout laminae I–IIo, approximately one third of c-Fos were colocalized with *Grpr*^*tdTom*^ neurons (Fig. 5a, b), suggesting the involvement of GRPR neurons in mechanical itch transmission. To functionally test the role of GRPR neurons, we examined the effect of the spinal ablation of GRPR neurons on mechanical itch using BB-sap (500 ng) approach^22^. The ablation of GRPR neurons was confirmed by the lack of scratching response to CQ (Fig. 5d). BB-sap treatment almost abolished mechanical itch (Fig. 5c and Supplementary movie 2). A comparison of different approaches suggests that the dose of BB-sap (400 ng) used by previous studies is likely to be too low, as mice still showed substantial scratching bouts to CQ injection^21,26,27^, suggesting a partial ablation of GRPR neurons (Supplementary Table 1). To test this possibility, we repeated the ablation test using BB-sap (400 ng). Interestingly, we found that mechanical itch is normal even though mice treated with BB-sap showed only approximately 20 scratching bouts to CQ (Supplementary Fig. 4a, b and Table 1). Only when mice treated with BB-sap scratched <5 times did mice fail to show mechanical itch behavior (Supplementary Table 1). We also counted the number of *Grpr* neurons in spinal cord slices after blank-sap, BB-sap (400 ng), and BB-sap (500 ng) treatment, respectively. The results showed that the number of *Grpr* neurons was lowest after BB-sap 500 ng treatment (Supplementary Fig. 4c–f), suggesting a complete ablation of GRPR neurons by BB-sap 500 ng. Therefore, a key prerequisite for evaluating whether mechanical itch depends on GRPR neurons is to ablate GRPR neurons completely, manifesting in the absence of CQ-evoked scratching behavior^6^. Recent studies have shown that pharmacological activation of spinal NPY1R can inhibit both mechanical^27^ and chemical itch^28,29^. This promoted us to examine to what extent *Npy1r* and *Grpr or Tac2* are co-expressed in the spinal cord using RNAscope. We found that approximately 35% of *Grpr* neurons express *Npy1r*, whereas only 11% express *Tac2* (Supplementary Fig. 4g–j). These findings raise the possibility that NPY1R agonists or NPY may act on GRPR neurons via NPY1R to inhibit chemical itch.

**Fig. 6:**
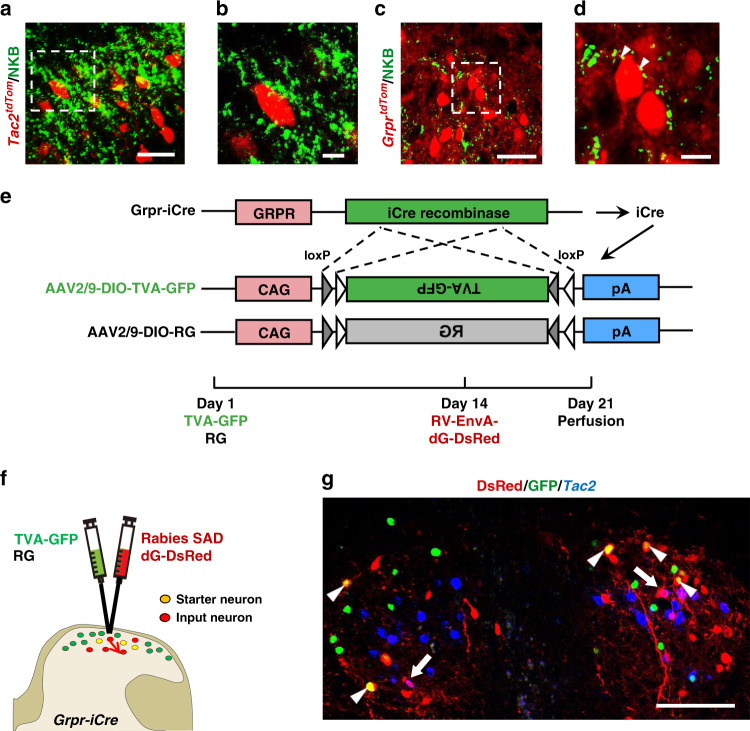
GRPR neurons form monosynaptic connections with *Tac2* neurons. **a**, **c** Representative IHC images of NKB staining (green) in the cervical spinal cord of *Tac2*^*tdTom*^ (red) mice (**a**) and *Grpr*^*tdTom*^ (red) mice (**c**). **b**, **d** High power images of the boxed areas in **a**, **c**, respectively. Scale bars, 20 μm in **a**, **c**; 5 μm in **b**, **d**. **e**, **f** Schematic of intraspinal injection of TVA-EGFP/RVG virus and RV-dG-dsRed virus. **g** Representative ISH image of *Tac2* (blue) in the cervical spinal cord after RV*dG* injections. Arrowheads indicate *Grpr*^*iCre*^ starter neurons (yellow) expressing GFP (green) and dsRed (red). Arrows indicate *Tac2*^+^ input neurons (dsRed^+^ and blue^+^) targeting starter neurons. *n* = 3 mice. Scale bar, 100 μm.

Next, we sought to determine the type of inputs that GRPR neurons may receive by recording the response of *Grpr*^*tdTom*^ neurons located in laminae I–IIo to the root stimulation. Using the same protocol for recording *Tac2*^*tdTom*^ neurons, we found that *Grpr*^*tdTom*^ neurons predominantly received polysynaptic Aβ input (71.4%, 30/42), whereas no monosynaptic Aβ input was detected (Fig. 5e–g). These data suggest that GRPR neurons are endowed with the capacity of receiving light touch information indirectly from *Tac2* neurons. To examine whether *Tac2* neurons may form synaptic contacts with GRPR neurons, we first performed NKB IHC in the cervical spinal cord of *Tac2*^*tdTom*^ mice (Fig. 6a, b) and *Grpr*^*tdTom*^ mice (Fig. 6c, d), respectively. Interestingly, the NKB staining was concentrated in laminae I–IIo where GRPR neurons are located (Fig. 6a–d). This unique distribution pattern of NKB implies that *Tac2* neurons project dorsally and their targets are located in laminae I–II, while some arborizing locally within the domain of laminae IIi–IIIo. Consistent with this view, numerous NKB punctate staining signals were detected surrounding and overlapping with *Grpr*^*tdTom*^ neurons (Fig. 6c, d). To examine whether *Tac2* neurons form monosynaptic contacts with GRPR neurons, we next employed rabies virus circuit tracing method^48^, using *Grpr*^*iCre*^ mice with glycoprotein-deleted rabies virus (RV*dG* virus) (Fig. 6e–g). The spinal cord of *Grpr*^*iCre*^ mice was injected with rAAV2/9- Ef1α-DIO- EGFP-TVA and rAAV2/9-Ef1α-DIO-RVG virus mix to label *Grpr*^*iCre*^ neurons with GFP and the glycoprotein of RV (RVG). Two weeks later, the RV-ENVA-dG-dsRed was injected into the same area to infect the GFP-labeled TVA-expressing *Grpr*^*iCre*^ neurons (yellow denotes starter neurons) (Fig. 6e, f). Assisted with glycoprotein in the starter neurons, RV*dG* would retrogradely label the input neurons with dsRed (Fig. 6f, g, red). Examination of *Tac2* expression (blue) with RNAscope found that the input neurons that targeted *Grpr* neurons express *Tac2* (Fig. 6g, arrows), indicating the existence of monosynaptic connections between *Tac2* neurons and GRPR neurons.

### *Tac2* neurons are required for alloknesis and dry skin itch

**Fig. 7:**
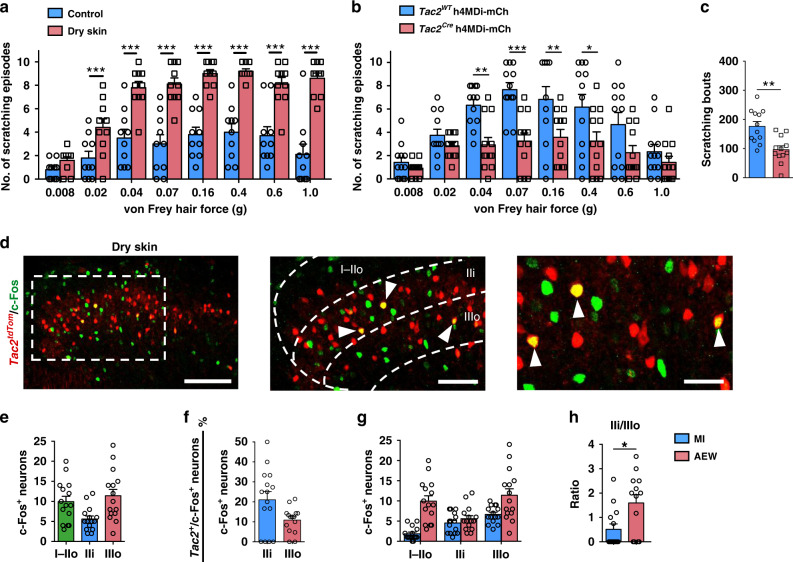
*Tac2* neurons are required for alloknesis and dry skin itch. **a** Increased mechanical itch in AEW-treated mice relative to the control. Two-way ANOVA with Bonferroni *post-hoc*. ***p = 0.00001, *n* = 10 mice per group. **b**, **c** Mechanical itch (**b**) and spontaneous scratching behavior (**c**) of AEW-treated mice after chemogenetic inhibition of spinal *Tac2*^*Cre*^ neurons. (**b** two-way ANOVA with Bonferroni *post-hoc*, *p = 0.027, **p = 0.005 for group 0.04 g, **p = 0.009 for group 0.16 g, ***p = 0.0001, *n* = 12 mice per group; **c** unpaired two-tailed Student’s *t-*test, **p = 0.001, *n* = 12 mice per group). **d** Representative IHC image of c-Fos (green) in the cervical spinal cord of AEW-treated *Tac2*^*tdTom*^ (red) mice. Higher magnification of the boxed area (middle), which is shown at higher magnification (right). Arrowheads indicate double c-Fos^+^/*Tac2*^*tdTom*+^ neurons. Scale bars, 100 μm in left of **d**; 50 μm in middle of **d**; 25 μm in right of **d**. **e** Quantification of c-Fos+ neurons in laminae I-IIo, IIi and IIIo, respectively. **f** The ratio of *Tac2*^*tdTom*^ and c-Fos double positive neurons in c-Fos positive neurons in laminae IIi and IIIo. **g** Comparison of the number of c-Fos^+^ neurons in laminae I-IIo, IIi and IIIo between control mice stimulated by von Frey hair (0.07 g) and AEW mice. **h** Comparison of the ratio of c-Fos^+^/ *Tac2*^*tdTom*+^ neurons in lamina IIi versus IIIo between control and AEW mice. *p = 0.016, unpaired two-tailed Student’s *t-*test, *n* = 3 mice per group. All data are presented as means ± s.e.m. and error bars represent s.e.m. Source data are provided as a Source Data file.

Next we asked why alloknesis is exacerbated under chronic itch condition. To ascertain whether *Tac2* neurons have a role in alloknesis associated with chronic itch, we employed a dry skin model using mice treated with acetone–ether–water (AEW)^42^, in which the loss of Piezo2-Merkel cell signaling contributed to alloknesis^31^. Mice treated with AEW displayed pronounced alloknesis compared to control mice without AEW treatment (Fig. 7a). Importantly, chemogenetic inhibition of *Tac2*^*Cre*^ neurons not only reversed enhanced alloknesis associated with dry skin itch (Fig. 7b), but also spontaneous scratching behavior, which reflects chemical itch induced by AEW treatment (Fig. 7c). In accordance with these findings, we found that c-Fos was induced in *Tac2*^*tdTom*^ neurons of mice treated with AEW in the absence of von Frey hair stimulation (Fig. 7d). Quantification of c-Fos^+^ and Tomato^+^/c-Fos^+^ double positive neurons indicates that while c-Fos appears to be evenly distributed across the dorsal horn of the spinal cord, more *Tac2*^*tdTom*^ neurons were activated by AEW treatment in lamina IIi than lamina IIIo (Fig. 7e–h). This contrasts with c-Fos pattern in naïve mice in response to mechanical itch stimulus (Fig. 1l, m).

**Fig. 8:**
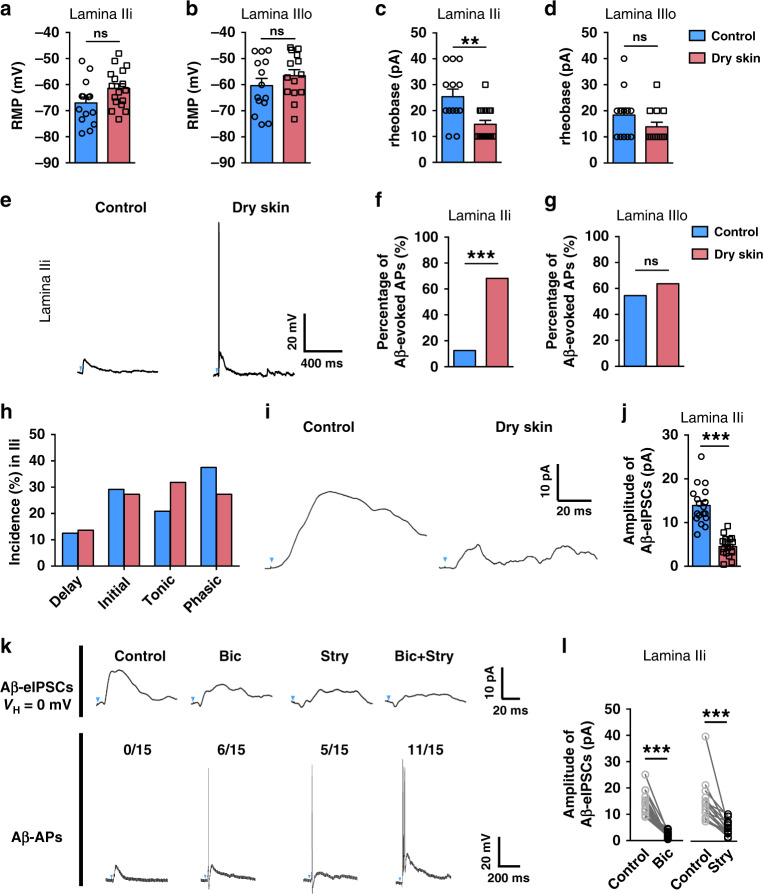
Increased excitability of *Tac2*^*tdTom*^ neurons of dry skin mice. **a**–**d** Resting membrane potential (RMP) (**a**, **b**) and the rheobase of action potential (**c**, **d**) for *Tac2*^*tdTom*^ neurons in lamina IIi (**a**, **c)** and lamina IIIo (**b**, **d**). Control: *n* = 14 neurons in **a**, **b**, 13 neurons in **c** and 12 neurons in **d**. Dry skin: *n* = 17 neurons in **a**, **c**, 14 neurons in **b** and 13 neurons in **d**. Two-tailed Mann–Whitney test. **p = 0.0041, ns not significant. Data were from three mice per group. **e** Representative traces for Aβ-evoked APs in *Tac2*^*tdTom*^ neurons in laminae IIi of control mice (left) and dry skin mice (right). **f**, **g** Percentage of Aβ-evoked APs in *Tac2*^*tdTom*^ neurons in laminae IIi (**f**) and IIIo (**g**) of control and dry skin mice, respectively. Control: *n* = 24 neurons in **f**, *n* = 11 neurons in **g**; dry skin: *n* = 22 neurons in **f**, *n* = 11 neurons in **g**, Chi-square test, ***p = 0.0001, ns not significant. Data from three mice per group. **h** Firing patterns of *Tac2*^*tdTom*^ neurons in control mice (*n* = 24 neurons) and dry skin mice (*n* = 22 neurons). Data from five mice per group. **i** Representative traces for Aβ-eIPSCs in *Tac2*^*tdTom*^ neurons in lamina IIi of control mice (left) and dry skin mice (right). **j** The amplitude of Aβ-eIPSCs on *Tac2*^*tdTom*^ neurons in lamina IIi of control mice and dry skin mice. ***p = 0.00001, two-tailed unpaired Student’s *t-*test, *n* = 18 neurons per group. **k** Representative traces of Aβ-eIPSCs on *Tac2*^*tdTom*^ neurons in lamina IIi before (control) and after bath application of bicuculline (Bic) and/or strychnine (Stry). Blue arrows indicate stimulation artifacts. Data were from three mice per group. **l** The amplitude of Aβ-eIPSCs on lamina IIi *Tac2*^*tdTom*^ neurons before (control) and after bath application of bicuculline or strychnine. ***p = 0.0001, two-tailed paired Student’s *t-*test, *n* = 15 neurons per group. Data are presented as means ± s.e.m. Error bars represent s.e.m. Source data are provided as a Source Data file.

Finally, we tested whether *Tac2* neurons in lamina IIi and IIIo are differentially activated in mice with dry skin itch by examining the excitability and Aβ-evoked action potentials (Aβ-APs) of *Tac2*^*tdTom*^ neurons of mice treated with AEW. While resting membrane potentials (RMP) remained unchanged (Fig. 8a, b), the rheobase of action potentials of *Tac2*^*tdTom*^ neurons in lamina IIi decreased significantly compared to the control mice (Fig. 8c, 25.4 pA vs. 14.7 pA). In contrast, no such changes were observed for *Tac2*^*tdTom*^ neurons in lamina IIIo (Fig. 8d), indicating that *Tac2*^*tdTom*^ neurons in lamina IIi are sensitized under dry skin itch condition. However, the firing patterns of *Tac2*^*tdTom*^ neurons in lamina IIi were unchanged in dry skin mice (Fig. 8h). Consistently, increased Aβ-APs were found exclusively in lamina IIi rather than lamina IIIo *Tac2*^*tdTom*^ neurons under dry skin condition (Fig. 8e–g). Furthermore, the Aβ-induced synaptic inhibition (eIPSC) was significantly reduced in dry skin itch mice (Fig. 8i, j). To further examine the synaptic inhibition on *Tac2*^*tdTom*^ neurons in lamina IIi, we tested the effect of GABA_A_Rs antagonist and GlyRs antagonist on Aβ-APs^47^. Of 15 *Tac2*^*tdTom*^ neurons in lamina IIi without Aβ-APs, 11 showed Aβ-APs after bath application of bicuculline and strychnine (Fig. 8k). These results suggest that *Tac2*^*tdTom*^ neurons in lamina IIi with Aβ inputs are modulated by GABA/glycine-dependent feedforward inhibition (Fig. 8k, l).

## Discussion

How innocuous tactile information is converted into irritating itch sensation is a fascinating question. In this study, we have used a combination of molecular, pharmacological, electrophysiological, chemogenetic, intersectional genetic ablation, and monosynaptic neural circuit tracing approaches to identify *Tac2* neurons, which are exclusively located in laminae IIi–IIIo of the spinal cord as a principal neural circuit for mechanical itch. Importantly, we demonstrate that GRPR neurons are an integral component of the circuitry for touch-to-itch conversion. Notably, we found that the behavior of *Tac2*^*tdTom*^ neurons is similar to *Ucn3*^*tdTom*^ neurons located in LTMR-RZ, which have recently been implicated in mechanical itch transmission^26^, under both naïve and dry skin conditions (Supplementary Table 2). This suggests that *Tac2* neurons are a subpopulation of *Ucn3*^*tdTom*^ neurons in laminae II–III. Given that *Ucn3*^*tdTom*^ neurons are distributed throughout laminae I–III, resulting from tracking transient expression of *Ucn3* during postnatal development^26^, we argue that *Tac2* neurons, which are readily identifiable anatomically in the adult spinal cord, represent a bona fide neural circuit for mechanical itch.

The loss of mechanical itch in mice treated with BB-sap suggests a crucial role of GRPR neurons in mechanical itch transmission. Anatomically, rabies virus circuit tracing that revealed monosynaptic connections between *Tac2* neurons and GRPR neurons supports this conclusion. The discrepancies between the present and previous studies^21,26,27^ can be ascribed to several methodological differences (see Supplementary Table 1). First, poking the nape of naïve mice ten times instead of five is advantageous (Supplementary Table 1), because it permits comparable evaluation of mechanical itch between naïve mice and mice treated with BB-sap using the same approach (nape). Second, given that application of von Frey to the nape evokes only a few scratching bouts (<10) per ten stimulations in naïve mice, a partial ablation of GRPR neurons, as manifested in approximately 50 scratches induced by pruritogens^21,27^, is insufficient for blocking mechanical itch. A key difference between our study and the others is the dose of BB-sap used. In our study, we found that 400 ng BB-sap is not sufficient to abolish mechanical itch. However, one caveat is that these different doses may not be absolute and have to be determined empirically by the users. This is because the activity and potency of BB-sap differ from lot to lot and additionally depend on the storage conditions by individual laboratories. We have found a gradual reduced activity of BB-sap over years and accordingly the dose of BB-sap must be adjusted accordingly to achieve complete ablation of GRPR neurons. Because it is difficult to evaluate subtle molecular differences between 400 and 500 ng in the spinal cord, perhaps the most convenient and simple way to verify the completeness of ablation of GRPR neurons is to check if CQ-evoked scratches are abolished after 2 or 3 weeks of injection. If not, additional dose of BB-sap should be injected to abate remaining GRPR neurons, which would ensure the loss of mechanical itch evoked from the nape. Fortunately, a relatively higher dose of BB-sap does not produce observable detrimental effect on mice. Could BB-sap at 500 ng or higher produce nonspecific effect that can be ascribed to the loss of mechanical itch? While this possibility cannot be excluded with certainty, it seems less likely. Most importantly, our finding is consistent with the observation that *Grpr *neurons express *Npy1r* (Supplementary Fig. 4g, h), as well as recent pharmacological studies showing that exogenous NPY or NPY1R agonist inhibit chemical itch^28,29^.

The present finding is further consistent with the observation of augmented alloknesis in a mouse model of dry skin itch^21,26,27,31,42^. One notable difference between human and mouse chronic itch models is that the latter is a type of chemical itch in nature, for the development and maintenance of scratching behavior are contingent on periodic application of chemicals to the nape as well as enhanced expression of GRP in DRGs and GRPR in the spinal cord^6,49,50^. Therefore, the fact that enhanced alloknesis in pathological dry skin itch in mice underscores that mechanical and chemical itch are inherently coupled, with the former depending on the latter. Moreover, since the perception and motor output of acute chemical itch, dry skin itch and mechanical itch are identical, it is economic for spinal GRPR neurons to convert touch to itch rather than using a separate neural pathway for mechanical itch transmission. Given that histaminergic itch transmitted via NMB and NMBR neurons is also converged on GRPR neurons^23^, GRPR neurons are the last interneuron station in the spinal cord for integrating and transmitting mechanical itch from the periphery to the brain^9,51^.

**Fig. 9:**
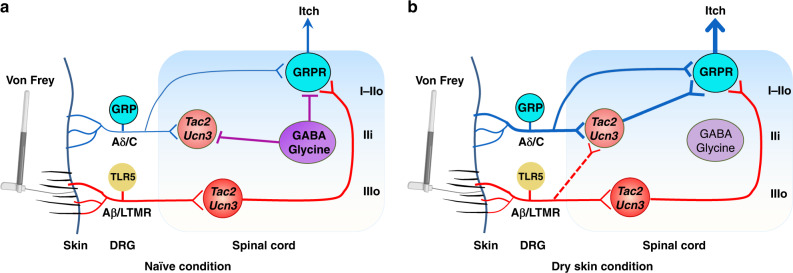
Schematics showing the spinal neural circuitry for touch-to-itch conversion. **a** Under naive condition, *Tac2/Ucn3*^*tdTom*^ neurons in lamina IIIo receive innocuous light touch information via LTMR Aβ/TLR5 fibers directly and in turn relay the touch information to GRPR neurons in laminae I–IIo, which convert it to itch. By contrast, *Tac2/Ucn3*^*tdTom*^ neurons in lamina IIi which receive direct inputs from C/Aδ fibers and indirect Aβ inputs may remain silent or inactive resulting from feedforward inhibition of GABAergic/glycinergic neurons. **b** Under dry skin itch condition, GRP primary afferents convey chemical itch information via GRP to GRPR neurons directly. In addition, enhanced chemical itch relayed by C/Aδ fibers recruit IIi *Tac2/Ucn3*^*tdTom*^ neurons to relay chemical itch information to GRPR neurons. Light touch information conveyed by *Tac2/Ucn3*^*tdTom*^ neurons, along with newly recruited IIi *Tac2/Ucn3*^*tdTom*^ neurons, and chemical itch converge on GRPR neurons. Augmented activity of GRPR function can also be resultant from reduced or a loss of feedforward inhibition mediated by GABAergic/glycinergic neurons, giving rise to exacerbated chronic itch and alloknesis.

Our analysis reveals that lamina IIi and IIIo *Tac2* neurons differ in firing patterns and types of input received. While approximately 28.0% of IIi *Tac2* neurons can receive polysynaptic Aβ fiber input, they primarily receive C/Aδ fiber input with negligible monosynaptic Aβ fiber input. By contrast, approximately half of IIIo *Tac2* neurons receives monosynaptic Aβ-LTMR input, making them best suited for transmitting innocuous touch information directly. Consistently, a significantly higher percentage of *Tac2* neurons in lamina IIIo than IIi generated Aβ evoked action potentials in naïve mice. The finding of two discrete subpopulations of *Tac2* neurons provides a neuroanatomic basis for explaining why *Tac2* neurons transmit mechanical but not chemical itch under naïve conditions. It is conceivable that lamina IIi *Tac2* neurons may be inactive or “silent” in naïve conditions. The observation of a reduction of feedforward inhibition mediated by GABA/glycine for IIi *Tac2* neurons under a dry skin condition, manifested by increased incidence of Aβ-evoked APs and higher intrinsic excitability, suggests that they are more active and sensitized relative to normal conditions. Therefore, one plausible explanation may be that IIi *Tac2* neurons are recruited from their silent state to transmit dry skin chemical itch directly via C/Aδ fibers and/or mechanical itch indirectly via Aβ fibers. This may give rise to exaggerated alloknesis associated with dry skin itch condition (Fig. 9). It is also possible that an increased excitability of GRPR neurons under chronic itch conditions^47,49^ concurrently contributes to alloknesis. Taken together, touch-evoked alloknesis appears to show laminar-specific and translaminar-specific modular connectivity that to some degree resembles touch-induced allodynia in naïve and pathological conditions (Fig. 9)^41,52,53^.

In view of the present findings that *Npy1r* is expressed in GRPR neurons (Supplementary Fig. 4g, h), it is conceivable that exogenous administration of NPY or NPY1R agonists in the spinal cord can activate NPY1R to inhibit the function of GRPR neurons and thus inhibit both chemical and mechanical itch^28,29^. Since not all GRPR neurons express NPY1R, the inhibitory effect of NPY or NPY1R agonists on chemical itch could depend on the type of pruritogens or subtypes of GRPR neurons expressing NPY1R. Despite these studies, it seems less likely that NPY–NPY1R signaling, or inhibitory neural circuits in general, is involved in mechanical itch inhibition endogenously for several reasons. Firstly, analogously to acute pain stimuli to which animals respond with withdrawal behavior, innocuous light touch does not penetrate the skin. To protect from a potential harm, withdrawal or light scratching/wiping is sufficient to distant from or remove trivial irritants on the skin. By contrast, activating an endogenous inhibitory neural circuit usually requires more intense scratching behavior. Indeed, once the von Frey hair is removed, mice no longer scratch the spot being touched. Consistent with this notion, ablation or inhibition of various populations of spinal inhibitory neurons does not impair acute thermal and mechanical pain behaviors^21,54,55^.

Secondly, GABA/glycine are adequate for regulating *Tac2*/*Ucn3*^*tdTom*^ neurons through feedforward inhibition via GABAergic/glycinergic neurons^26^. Because mechanical itch travels through GRPR neurons, which are directly subject to GABA/glycine-mediated inhibition of galanin neurons^54,56,57^, the physiological relevance of inhibitory regulation of *Tac2*/*Ucn3*^*tdTom*^ neurons is currently unclear. It is possible that *Tac2*/*Ucn3*^*tdTom*^ neurons are kept in a quiescent state under an inhibitory control, which may explain why mechanical itch is rather infrequently experienced. Nonetheless, caution is warranted while extrapolating the endogenous function of a receptor from behaviors resulting from its pharmacological activation or inhibition, which could trigger a novel signaling pathway that may not occur in vivo^47^. On the other hand, there is substantial evidence that the spinal NPY–NPY receptor system plays a role in inhibition of nociceptive transmission under normal and neuropathic conditions^58–62^. Thus, it is not surprising that an artificial activation of spinal NPY neurons could inhibit somatosensory transmission across modalities. Conversely, excessive scratching/biting/licking behaviors caused by ablation of spinal NPY neurons, which previously were interpreted as mechanical itch^21,27^, might alternatively be suggestive of mechanical hypersensitivity resulting from a loss of the tonic inhibition mediated by GABAergic neurons that express NPY^63^. Indeed, mice treated with intrathecal administration of a NPY2R antagonist exhibited robust pain- but not itch-related scratching behavior, suggesting a role of NPY signaling in gating spontaneous pain^61^. Accordingly, von Frey stimulation applied to the nape or cheek may evoke pain-related scratching behavior due to mechanical hypersensitivity caused by the ablation of NPY neurons. While not tested, one can predict that galanin neurons, which mediate direct inhibition of GRPR neurons^54^, may also be important for gating mechanical itch. Collectively, these data suggest that mechanical itch can be simultaneously gated at the level of *Tac2* neurons and GRPR neurons (Fig. 9).

In summary, our results show that lamina IIIo *Tac2* neurons function as a key entry node for receiving and encoding innocuous touch, which is accessible to GRPR neurons via the *Tac2*–GRPR monosynaptic neuronal connection. GRPR neurons subserve as convergent and integrating circuit for chemical and mechanical itch and represent the last output station from the spinal cord to the brain (Fig. 9)^9^. Under pathological itch conditions, lamina IIi *Tac2* and laminae I–IIo GRPR neurons might be sensitized to convey heightened alloknesis, in part due to disinhibition, thereby exacerbating chronic itch conditions (Fig. 9). Hence, our study suggests a previously unknown spinal circuitry that is exquisitely hard-wired for touch-to-itch conversion.

## Methods

### Animals

Experiments were carried out on C57BL/6J (Stock no.000664, Jax mice), *Tac2*^*Cre*^ mice^33^, *Grpr*^*iCre*^ mice^47^, Ai32 mice (Stock no. 024109, Jax mice), Ai9 mice (Stock no.007909, Jax mice), *Lbx1*^*flpo*^ mice^41^, *Tau*^*DTR*^ mice^41^ and their wild-type littermates unless indicated otherwise. All mice were housed under a 12 h light/dark cycle. Mice were housed in clear plastic cages with no more than 5 mice per cage in a controlled environment at a constant temperature of ~23 °C and humidity of 50 ± 10% with food and water available ad libitum. Male animals of 2–3 months of age were used in the experiments. All experiments conformed to guidelines set by the National Institutes of Health and the International Association for the Study of Pain and were reviewed and approved by the Institutional *Animal* Care and Use *Committee* (IACU C) at Washington University School of Medicine.

### Acute pain behavior test

Mice should have 3 days of acclimation in all the acute pain behavior tests. Mechanical sensitivity was assessed using a set of calibrated von Frey filaments. The lateral plantar surfaces of the hindpaw were stimulated with defined von Frey filaments for five times with 10-s intervals. The smallest filament that evoked reflexive flinches of the paw on three of the five trials was taken as the paw withdrawal threshold. To measure tail flick threshold to noxious mechanical stimulation, a Randall–Selitto Analgesy-meter was used. This instrument generates a mechanical force that increased linearly with time. Mice were held gently, and the force was applied directly to the dorsal surface of the tail 2.5 cm from its end via a cone-shaped plunger. The tail flick threshold is defined as the average force of five trials with 10-min intervals, in grams, at which the mouse attempts to flick its tail (cut-off force 250 g). Thermal sensitivity was determined by Hargreaves test, hot plate and tail flick tests. For Hargreaves test, the plantar paw surface was exposed to a beam of radiant heat with 10-min intervals. The paw withdrawal latency was tested five times per animal and averaged for analysis. For hot plate test, the latency for the mouse to lick its hindpaw or jump from the hotplate (48, 52, and 56 °C) was recorded. For the tail flick test, the end of tail was exposed to a beam of radiant heat with 10-min intervals. The tail flicking latency was tested five times per animal and averaged for analysis.

### Rotarod test

Mice were placed on a rotarod apparatus that accelerates 5–20 revolution per minute (r.p.m.) for 5 min and trained to maintain its belaying walking on the first two days. On the third day, rod accelerated 5–40 r.p.m. and mice were tested three times with 10-min intervals (cut-off time 300 s). The latencies of mice to fall off were recorded for analysis.

### Acute itch test

Scratching behaviors were performed by injections of chloroquine (CQ) (Sigma, Cat. No. C6628)^22^. Briefly, the injection area was shaved at least 3 days before experiments. Prior to the experiments, each mouse was placed in a plastic arena (10 × 11 × 15 cm) for 30 min to acclimate. Mice were briefly removed from the chamber and intradermally (i.d.) injected at the nape with CQ at the dose of 200 µg in 50 µl saline. Hind limb scratching behavior towards the injected area was counted by observers blinded to the group or genotype of the mice.

### Bombesin-saporin treatment

Blank-saporin or bombesin-saporin (Advanced Targeting) was intrathecally (i.t.) injected at 500 or 400 ng per 10 µl 14 days before behavioral tests.

### Dry skin itch

The dry skin model was implemented as described^49^. Briefly, the nape of mice shaved at least 3 days before experiments. A mixture of acetone (Sigma, Cat. No. 179124) and diethylether (Sigma, Cat. No. 309966) (1:1) was painted on the neck skin for 15 s, followed immediately by a 30-s of distilled water application (AEW). This regiment was administrated twice daily for 5–7 days. Littermate control mice received water only for 45 s on the same schedule. Spontaneous scratches were examined for 60 min in the early morning on day 5–7.

### Mechanical itch or alloknesis test

The nape of mice was shaved at least 3 days before experiments. Mice were acclimated in a recording chamber (20 × 10 × 12.5 cm) for 3 days. Mechanical stimuli on the nape were delivered with von Frey filaments ranging from 0.008 to 1.0 g. The von Frey filament was held for up to 1 s or until the mice responded. Positive responses were counted as hindlimb scratching towards the site of mechanical stimulation. Each von Frey filament was tested ten times on different random points of the nape with 10-s intervals. For AEW mice, mechanical stimuli were delivered on the border of the AEW-treated area. The number of scratching episodes for each filament were plotted for comparison.

### C-Fos induction

The nape of the mice was shaved at least 3 days before experiments. Mice were acclimated in the experimental chambers for 3 days. On the day for c-Fos induction, all mice were gently put into the chambers and acclimated for 2 h. Free ambulating mice were not given any stimulations. For chemical itch stimulus, mice were gently removed from the chamber and anesthetized by isoflurane. CQ (200 µg in 50 μl saline) was i.d. injected at the right nape of the mice. Then the mice were returned to the chamber for recovery. For mechanical dynamic stimulus, mice were brushed at 2 cm s^−1^ in nape area for 30 min. For mechanical pain stimulus, the lateral plantar surface of the right hindpaw was stimulated with 1.4 g von Frey filament 90 times within 30 min with 20-s intervals. For mechanical itch stimulus, the right nape was stimulated with 0.07 g von Frey hair 20 times with 10-s intervals. For optogenetic stimulus, the mice received blue light stimuli (473 nm) with 30-s on and 270-s off for six trials in 30 min. For chemogenetic activation, mice were i.p. injected with clozapine (0.1 mg kg^−1^) under anesthesia state by isoflurane. After 90 min, mice were perfused for c-Fos immunohistochemistry (IHC). For von Frey-hindpaw group, the lumbar spinal cords were used. For all the other groups, the cervical spinal cords were used.

### Immunohistochemistry

Mice were deeply anesthetized with isoflurane and perfused intracardially with 4% paraformaldehyde in PBS^64^. Tissues were dissected, post-fixed for 8 h, and cryoprotected in 20% sucrose overnight at 4 °C. Free-floating frozen sections were blocked in a 0.01 M PBS containing 2% donkey serum and 0.1% Triton X-100 followed by incubation with primary antibodies overnight at 4 °C and secondary antibodies for 2 h at room temperature. Sections were mounted with FluoromountG (Southern Biotech). The following primary antibodies were used: chicken anti-GFP (1:500, Aves Labs, GFP-1020), rabbit anti-c-Fos (1:4000, Abcam, ab190289), rabbit anti-lmx1b (1:500, Invitrogen, Cat.PA5-34471), rabbit anti-Pax2 (1:500, Invitrogen, Cat.71-6000) and rabbit anti-NKB (1:500, Novus, Cat.NB300-201). The following secondary antibodies were used: Alexa-Fluor 488 conjugated donkey anti-chicken (1:500, Jackson ImmunoResearch, 703-545-155), Cy3-conjugated donkey anti-rabbit (1:500, Jackson ImmunoResearch, 711-165-152) and 488-conjugated donkey anti-rabbit (1:500, Jackson ImmunoResearch, 711-545-152). Fluorescent Images were taken using a Nikon C2+ confocal microscope system (Nikon Instruments, Inc.).

### Spinal fiber optic implantation

Mice were anesthetized with ketamine (90 mg kg^−1^) and xylazine (10 mg kg^−1^) cocktail. An incision of the cervical skin was made along the midline of the spine. The spinal column was fixed in a stereotaxic frame using spinal adapters (Stoelting, Cat. No. 51690). The spinal cord was exposed by removal of tissue around and between the vertebrae. A small burr hole was drilled ∼0.5–0.8 mm lateral from the midline to one side of the vertebra C3 or C4. A custom-made ferrule with ~0.25 mm fiber optic tip (200 µm in core diameter, Doric Lenses) was placed at the burr hole using a stereotactic holder and super glue gel with accelerant^65^. Dental cement (Lang Dental) was used to secure the fiber-optic ferrule onto the vertebra and the skin was closed with nylon sutures. Animals recovered in the home cage for 2 weeks before experiments.

### Optogenetic stimulation behavior

*Tac2*^*ChR2*^ mice and wild-type littermates (*Tac2*^*WT*^) were used for optical stimulation experiments. One day prior to the experiments, each mouse was placed in a plastic home cage (27 × 16.5 × 12.5 cm) for 30 min to acclimate. For stimulation, the fiber optic ferrule spinal implant was connected via a ferrule sleeve to a fiber optic cable with commutator (Doric Lenses) that was attached to a fiber-coupled 473 nm blue laser (BL473T8-150FC, Shanghai Laser and Optics Co.) with an ADR-800A adjustable power supply. The animal was allowed to acclimate being tethered to the cable for 30 min prior to stimulation. Laser power output from the fiber optic was measured using a photometer (Thor Labs) and set to 10 mW from the fiber tip (fiber implants were tested and % efficiencies of power was recorded prior to implantation to ensure 10 mW final power from tips). An Arduino UNO Rev 3 circuit board (Arduino) was programmed and attached to the laser via a BNC input to control the frequency and timing of the stimulation. For 30-s stimulation, three trials (30-s on, 270-s off) were performed for each frequency (1, 5, 10, 20 or 30 Hz) with 1-day break between each frequency. For morphine injection, morphine (0.3 nmol in 10 μl saline) was i.t. injected 30 min before stimulation. Control mice were i.t. injected with saline. The mean value of the three trials for behavior responses was used in the results and analysis. Mice were recorded with a video camera from a side angle and played back on computer for assessments of the number of scratches by observers blinded to the animal groups.

### Intraspinal virus injection and chemogenetics

*Tac2*^*Cre*^ mice were anesthetized and the cervical vertebrae were exposed at C2–C6, while lumber vertebrae were exposed at L3–L5, and the vertebral column was mounted onto a stereotaxic frame with spinal adapters^47^. AAV8-Syn-DIO-hM4Di-mCherry (2.0 × 10^13^ vg ml^−1^, AddGene, Cat. No. 44362-AAVrg) was injected into the spinal cord bilaterally at six sites between successive vertebrae at C3–C4–C5 or injected into the left side of the spinal cord at three sites between successive vertebrae at L3–L4–L5, with a Hamilton Neuros-syringe with beveled needle (catalog number: 65458-02, 34-gauge, 20-degree angle). The syringe needle was inserted into the dorsal spinal cord vertically at a depth of ~500 μm to target the laminae II–III. The AAV was injected (~500 nl AAV per injection) at a rate of 50 nl min^−1^ with a Stoelting Quintessential Injector (QSI, catalog number: 53311) and the needle was slowly removed 10 min after the injection was complete. Three weeks were allowed for virus expression before clozapine injection. For chemogenetic experiments, clozapine (0.1 mg kg^−1^, i.p. injection) was used followed by behavioral tests 2 h after injection.

### Intersectional genetic ablation of *Tac2* neurons in the spinal cord

Triple mouse line (*Tac2*^*Cre*^, *Lbx1*^*flpo*^ and *Tau*^*DTR*^ mice) was generated for cell ablation. To ablate *Tac2* neurons in the spinal cord, i.p. injection of diphtheria toxin (DTX, 40 μg kg^−1^, List Biological Lab) was injected at day 1 and day 4, respectively^41^. Behavioral and histochemical experiments were performed 2 weeks after the second DTX injection.

### RNAscope in situ hybridization (ISH)

The spinal cord sections were processed according to the manufacturer’s instructions in the RNAscope Fluorescent Multiplex Assay v2 manual for fixed frozen tissue (Advanced Cell Diagnostics), and coverslipped with Fluoromount-G antifade reagent (Southern Biotech) with DAPI (Molecular Probes)^47,66^. The following probes, purchased from Advanced Cell Diagnostics were used: *Grpr* (nucleotide target region 463-1596; GenBank: NM_008177.2), *Tac2* (nucleotide target region 15-684; GenBank: NM_009312.2), *Vglut2* (nucleotide target region 1986-2998; GenBank: NM_080853.3), *Vgat* (nucleotide target region 894-2037; GenBank: NM_009508.2), and *Npy1r* (nucleotide target region: 227-1169; GenBank: NM_010934.4). Sections were subsequently imaged under a Nikon C2+ confocal microscope (Nikon Instruments, Inc.) in three channels with a 20× objective lens. Positive signals were identified as three punctate dots present in the nucleus and/or cytoplasm if the signal was shown up in small dots rather than filling up entire neurons. For co-localization studies, dots associated with single DAPI stained nuclei were assessed as being co-localized. Cell counting was done by a person who was blinded to the experimental design.

### Monosynaptic retrograde tracing

Briefly, 400 nl of the mixture of rAAV2/9-Ef1α-DIO-EGFP-TVA and rAAV2/9-Ef1α-DIO-RVG (2.0 × 10^12^ vg mL^−1^, BrainVTA Co., Ltd., Wuhan, China) (volume ratio: 1:1,) was injected into the cervical spinal cord of *Grpr*^*iCre*^ mice. Two weeks later, the spinal cord of mice was injected with 200 nl of RV-ENVA-dG-dsRed (2.0 × 10^8^ IFU mL^−1^, BrainVTA Co., Ltd., Wuhan, China) at the same site. One week later, the mice were perfused, and the spinal cord was sectioned and imaged under a Nikon C2+ confocal microscope (Nikon Instruments, Inc.). The sections were then used for RNAscope ISH using *Tac2* probe (Advanced Cell Diagnostics) and imaged under the confocal microscope. Images taken before and after ISH were aligned and merged for analysis.

### Electrophysiology

Adult mice (8–10 weeks old) were deeply anaesthetized with ketamine cocktail (ketamine, 90 mg kg^−1^ and xylazine, 10 mg kg^−1^). Then they were perfused with 30 ml ice cold (4 °C) NMDG slicing solution (in mM, 93 NMDG, 2.5 KCl, 1.25 NaH_2_PO_4_, 30 NaHCO_3_, 20 HEPES, 25 Dextrose, 12.1 N HCl, 5 Ascorbic acid, 2 Thiourea, 3 Na^+^ pyruvate, 10 MgSO_4_, 0.5 CaCl_2_, 12 N-acetylcysteine, pH was adjusted to 7.3–7.4 with NMDG). Spinal cord was isolated under oxygenated (95% O_2_, 5% CO_2_) sucrose-based dissection solution (in mM, 209 Sucrose, 2 KCl, 1.25 NaH_2_PO_4_, 5 MgCl_2_, 0.5 CaCl_2_, 26 NaHCO_3_, 10 Dextrose, pH was adjusted to 7.3–7.4) and the lumbar region was embedded in agar. Sections of the lumbar were obtained at 400 µm using a vibrating slicer (Vibratome 1000plus). The slices were recovered in a chamber containing 37 °C oxygenated holding solution (in mM, 92 NaCl, 2.5 KCl, 1.25 NaH_2_PO_4_, 30 NaHCO_3_, 20 HEPES, 25 Dextrose, 2 MgCl_2_, 2 CaCl_2_, pH was adjusted to 7.3–7.4) for 1 h.

Neurons were visualized with 593 nm light (TXRED filter) under an upright microscope (Olympus BX 51WI). Slices were mounted in a chamber (Warner RC 26 G) and perfused with oxygenated ACSF at 2 ml min^−1^ (in mM, 124 NaCl, 2.5 KCl, 1.25 NaH_2_PO_4_, 24 NaHCO_3_, 5 HEPES, 12.5 Dextrose, 1 MgCl_2_, 2 CaCl_2_, pH was adjusted to 7.3–7.4). Patch pipettes were pulled to a resistance of 6–8 MΩ. Signals were amplified with Multiclamp 700B and Digidata 1550 A and pClamp 10.7 software (Molecular Devices). Signals were filtered at 2 kHz and digitized at 10k Hz. Data were analyzed with Clampfit 10.7, Mini Analysis 6.0.1 (Synaptosoft) and Prism 7 software (GraphPad). Traces were plotted using Origin 2015 software (OriginLab).

For the root stimulation recording, the lumbar spinal cord of mice was removed, embedded and glued with the spinal cord midline parallel to the vibratome blade. Parasagittal sections of the lumbar cord were obtained at 500–550 µm^67^. 7–10 mm dorsal roots were kept. L4 or L5 root was sucked and injected currents by a suction electrode. *Tac2*^*tdTom*^ or *Grpr*^*tdTom*^ neurons were recorded by another electrode filled with normal pipette solution (in mM, 130 K gluconate, 10 NaCl, 0.2 EGTA, 10 HEPES, 1 MgATP, 5 Na_2_GTP, 1 MgCl_2_, pH was adjusted to 7.2). After establishing whole-cell configuration, the resting membrane potential was noted immediately. If the resting membrane potential was positive to −50 mV, the data were discarded. Evoked EPSCs were recorded from a holding potential of −70 mV, evoked IPSCs were recorded by holding membrane potential at 0 mV when eEPSCs were minimized^26^. Stimulus duration was 0.1 ms. Stimulus intensities were determined by performing extracellular recordings of compound action potentials from the dorsal root. The Discrete fibers were classified according to the following criteria: Aβ fibers (5–20 µA), LT/HT-Aδ (20–50 µA) and C fibers (100–500 µA)^68^. Neurons showing no failures at 20 Hz for Aβ, 2 Hz for Aδ, and 1 Hz for C were considered monosynaptic. Onset latencies varied <2 ms for monosynaptic A fiber mediated EPSCs. To examine feed-forward, bicuculline (10 μM, MilliporeSigma, St. Louis, MO) and/or strychnine (2 μM, MilliporeSigma, St. Louis, MO) were used to disinhibit the dorsal horn neurons^26^. Aβ-evoked IPSP, EPSP, or APs were detected by current clamp recording at the resting membrane potential.

### Statistics

Statistical methods are indicated when used. Values are reported as the mean ± standard error of the mean (s.e.m.). Statistical analyses were performed using Prism 7 (v7.0d, GraphPad, San Diego, CA). For parametric comparison between two group, an *F*-test was conducted to determine the similarity in the variances between the groups, and statistical significance was analyzed using the Student’s *t*-test. For multiple comparisons, Bartlett’s test for equal variances was used to determine the variances between the multiple groups and one-way or two-way analysis of variance (ANOVA) followed by *post hoc* test was used to test statistical significance. A *p* value of less than 0.05 was considered statistically significant.

### Reporting summary

Further information on research design is available in the Nature Research Reporting Summary linked to this article.

## Supplementary information

Supplementary Information

Reporting Summary

Description of Additional Supplementary Files

Supplementary Movie 1

Supplementary Movie 2

## Data Availability

The authors declare that all data supporting the findings of this study are available within the paper and its supplementary information files.

## Source data

Source Data
