# Supplementary material for: A spinal neural circuitry for converting touch to itch sensation
Source: Nat Commun. 2020 Oct 8;11:5074. doi: 10.1038/s41467-020-18895-7 (PMC7545208; doi:10.1038/s41467-020-18895-7)
Supplement: Supplementary file 1 — Supplementary Information [file 41467_2020_18895_MOESM1_ESM.pdf]

Supplementary Information

A spinal neural circuitry for converting touch to itch sensation

Chen et al.

This pdf file includes:

Supplementary Figure 1

Supplementary Figure 2

Supplementary Figure 3

Supplementary Figure 4

Supplementary Table 1

Supplementary Table 2

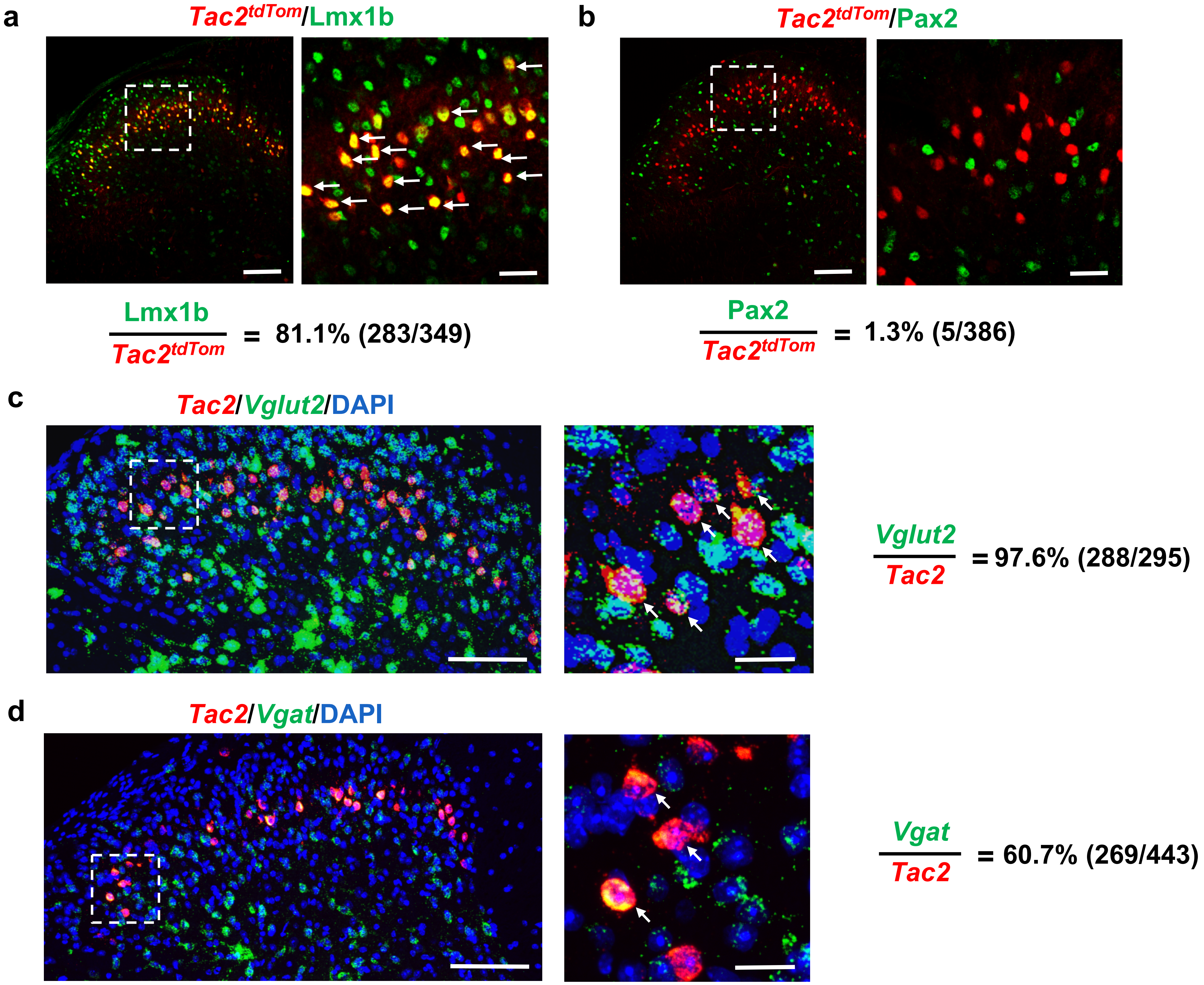

Supplementary Figure 1. Neurotransmitter phenotype of *Tac2*^{tdTom} neurons in the spinal cord. a, **b**, Representative images of Lmx1b (green) (**a**) and Pax2 (green) (**b**) immunofluorescence in the cervical spinal cord of *Tac2*^{tdTom} (red) mice. The white box indicates the area of higher magnification (right). The arrows denote Lmx1b^{+}/ *Tac2*$^{tdTom+}$ or Pax2^{+}/ *Tac2*$^{tdTom+}$ neurons. **c**, **d**, Representative images of *Vglut2* (green)(**c**) and *Vgat* (green)(**d**) with *Tac2* (red) by RNAscope in the cervical spinal cord of C57BL/6J mice. Nuclei is stained with DAPI (blue). The white box indicates the area of higher magnification (right). The arrows denote *Vglut2*$^{+}$/*Tac2*$^{+}$ and *Vgat*$^{+}$/*Tac2*$^{+}$ neurons. The percentage is calculated as double-positive neurons over total number of *Tac2*$^{tdTom+}$ or *Tac2*$^{+}$ neurons: Lmx1b (283/349), Pax2 (5/386), *Vglut2* (288/295), and *Vgat* (269/443). n = 9 sections from 3 mice. Scale bars, 100 µm in **a-d** (left); 25 µm in **a-d** (right).

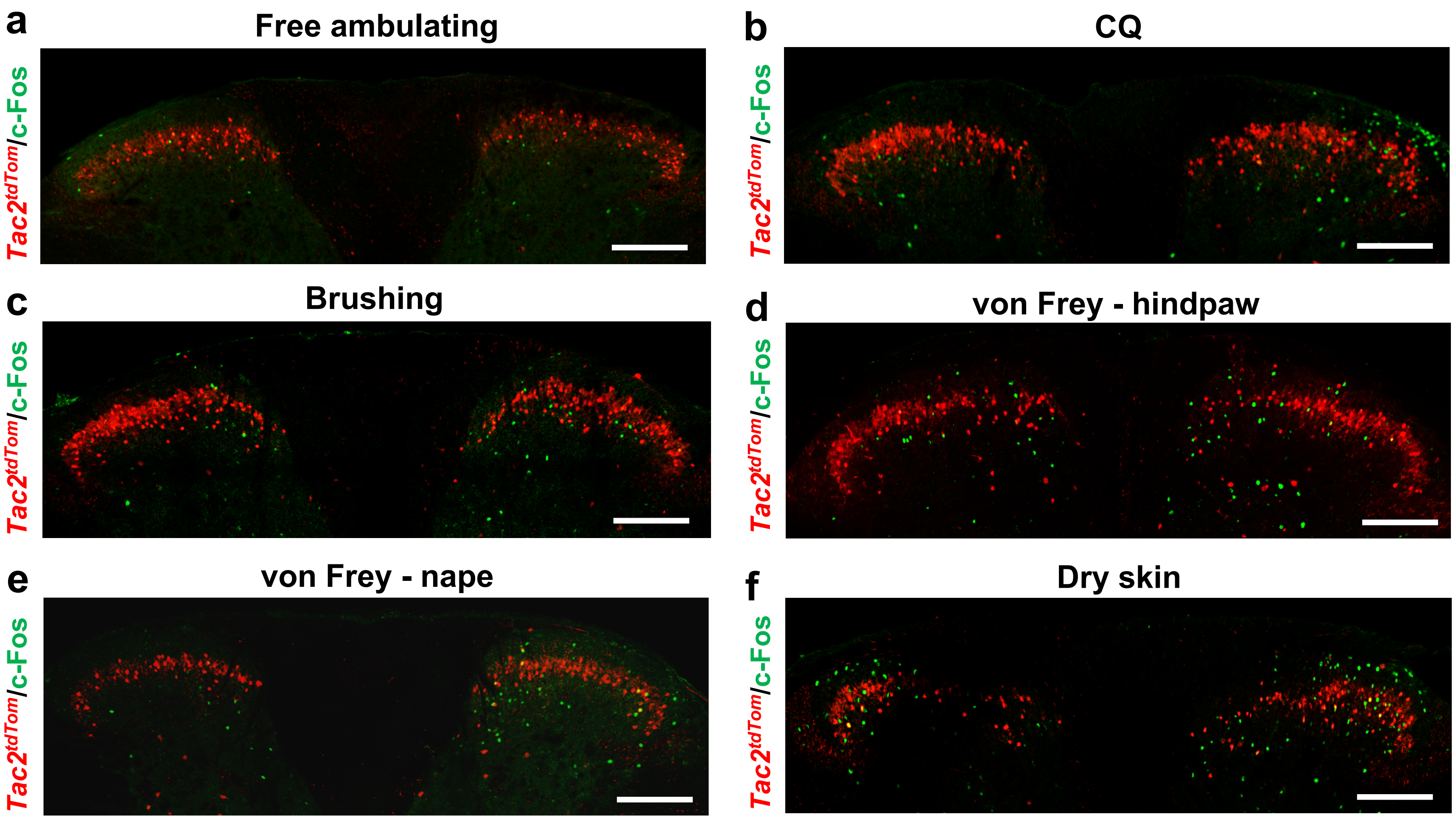

Supplementary Figure 2. *Tac2^Cre^* neurons are activated by mechanical itch stimulation and dry skin itch. a-f, Representative images of c-Fos expression (green) in the cervical cord of *Tac2^tdTom^* (red) of free ambulating mice (**a**), in response to i.d. CQ injection (**b**), brushing (**c**), von Frey hair stimulation in hindpaw (**d**), von Frey hair stimulation in nape (**e**) and AEW (**f**). n = 3 mice per group. Scale bars, 200 μm in **a**-**f**.

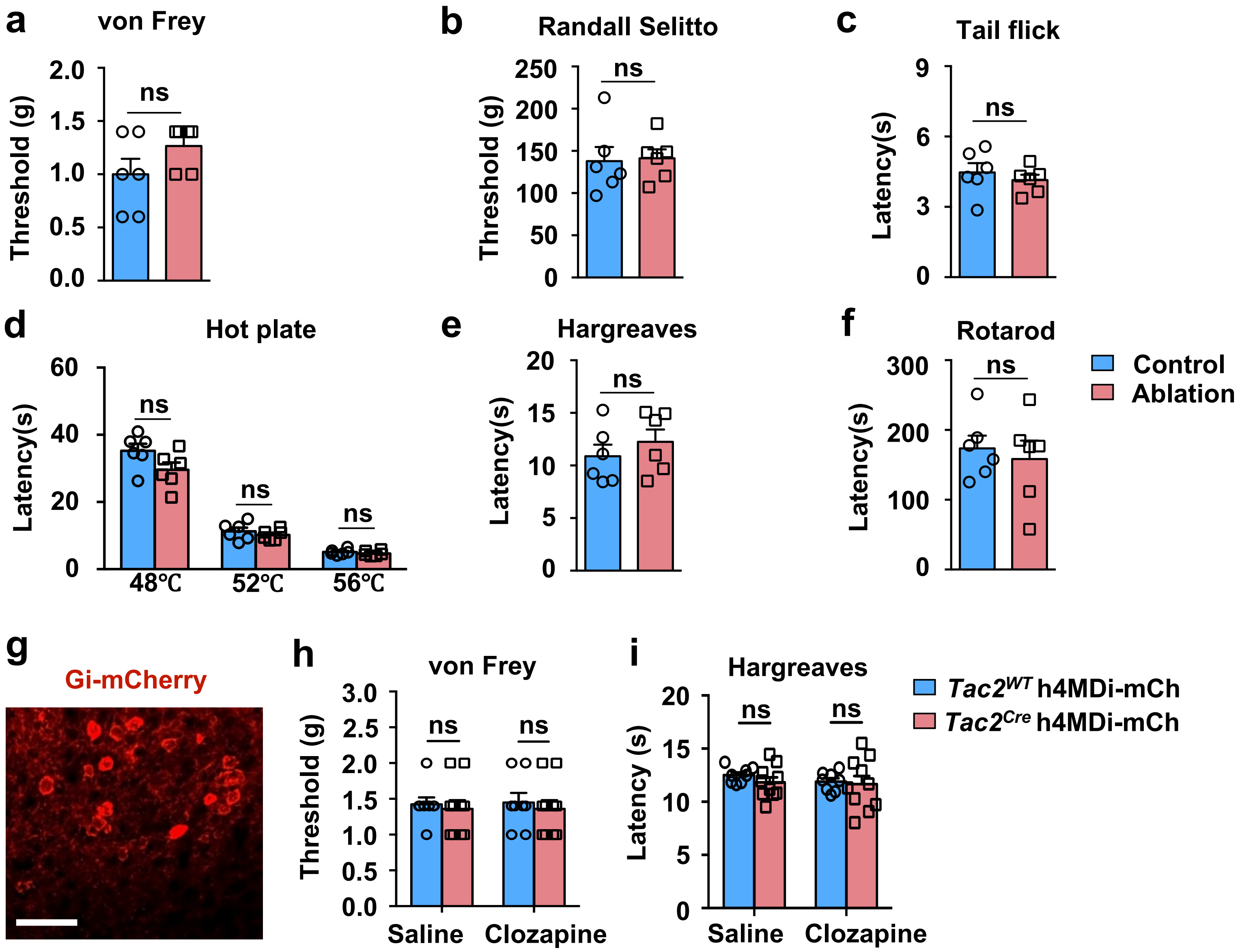

Supplementary Figure 3. Conditional ablation of spinal *Tac2*^{Cre} neurons did not affect pain or motor behaviors. a-f, Pain or motor behavior test in conditional ablation of *Tac2* neurons in spinal cord, including von Frey test **(a)**, Randall Selitto test **(b)**, Tail flick test **(c)**, Hot plate test **(d)**, Hargreaves test **(e)** and Rotarod test (**f**). (**a**-**c**, **e**, **f**; two-tailed Student's unpaired *t*-test, ns : not significant, n = 6 mice per group; **d**; two-way ANOVA with Bonferroni *post hoc*, ns : not significant, n = 6 mice per group). **g**, Representative IHC image of mCherry^{+} (red) neurons in the lumber spinal cord of *Tac2*^{Cre} mice infected with AAV8-hSyn-DIO-hM4Di (Gi)-mCherry virus. All 18 *Tac2*^{Cre} mice with virus injections were subjected for IHC verifications. Scale bar, 50 μm. **h**, **i**, Threshold of von Frey (**h**) and latency of Hargreaves (**i**) after chemogenetic inhibition of *Tac2*^{Cre} neurons in the lumbar spinal cord. Two-way ANOVA with Bonferroni *post hoc*, ns: not significant, n = 8 mice for *Tac2*^{WT} group and n = 10 mice for *Tac2*^{Cre} group. All data are presented as means ± s.e.m. and error bars represent s.e.m. Source data are provided as a Source Data file.

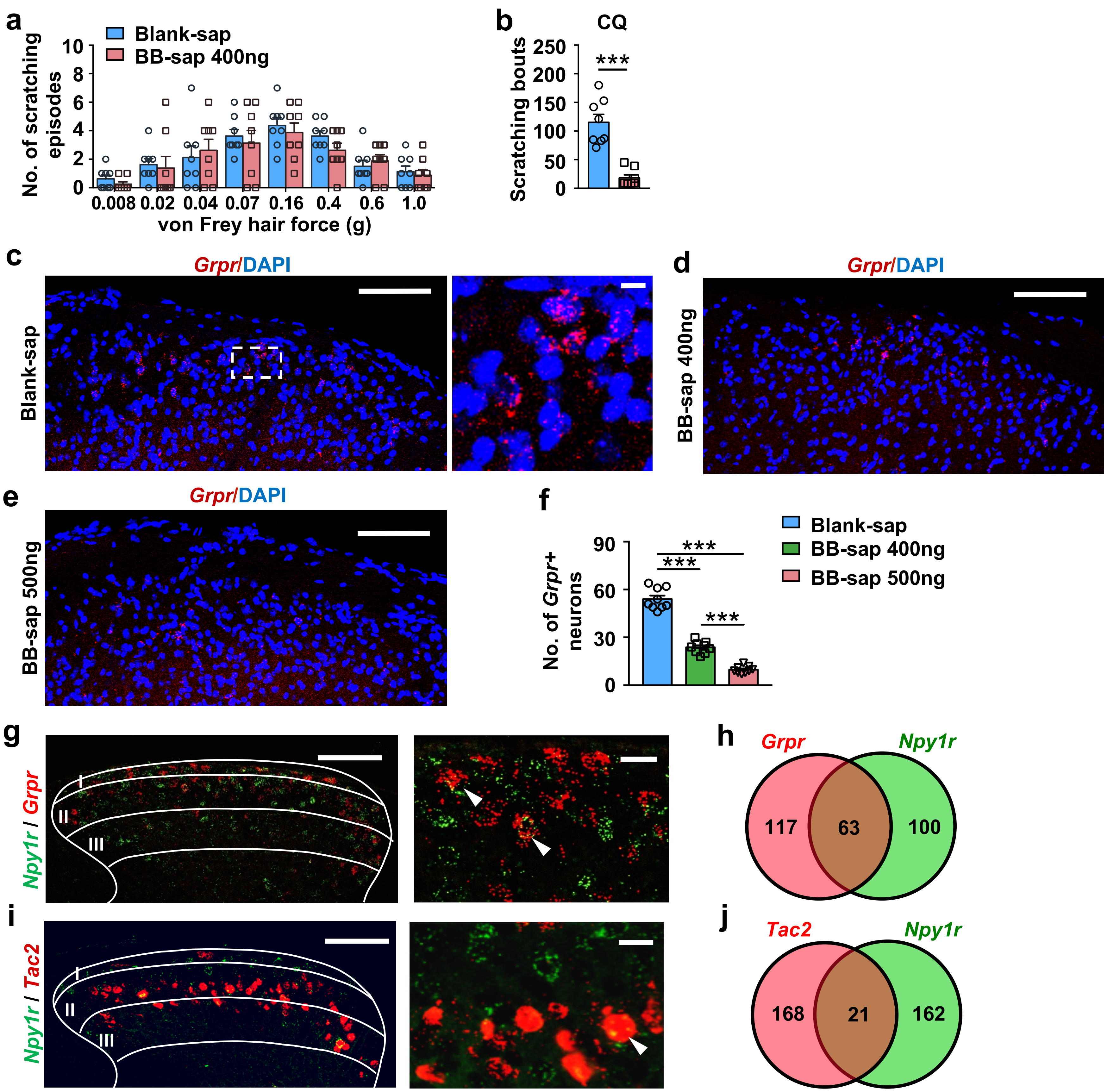

Supplementary Figure 4. Transmission of mechanical itch depends on GRPR neurons. **a**,**b**, Mechanical itch test (**a**) and CQ itch test (**b**) after BB-sap 400 ng treatment. (**a**; two-way ANOVA with Bonferroni *post hoc*, n = 10 mice per group; **b**; two-tailed Student's unpaired *t*-test, ***p = 0.00001, n = 10 mice per group). **c-e**, Representative RNAscope images of *Grpr* mRNA (red) in the cervical spinal cord of mice treated with blank-sap (**c**), BB-sap 400 ng (**d**), and BB-sap 500 ng (**e**). Scale bars, 100 μm. **f**, Comparison of number of *Grpr*$^+$ neurons among control, BB-sap 400 ng and 500 ng groups. One-way ANOVA with Tukey post hoc, ***p = 0.00001, n = 9 sections from 3 mice per group. **g-j**, RNAscope images in the superficial dorsal horn of the spinal cord (**g, i**) and Venn diagrams (**h, j**) showing co-expression of *Grpr* (red) (**g**,**h**) and *Tac2* (red) (**i**,**j**) with *Npy1r* (green). n = 9 sections from 3 mice per group. Arrow heads indicate co-expression neurons. Scale bars, 100 μm in left images and 20 μm in right images. All data are presented as means ± s.e.m. and error bars represent s.e.m. Source data are provided as a Source Data file.

Supplementary Table 1. Comparison of the methods for mechanical itch tests

Reference	Stimulation site		Stimulation times	Response percentage	Validation(BB-sap)		
					Dose	CQ 200 μg	Mechanical itch
Akiyama et al, 2012	Nape 7 mm away from injection site	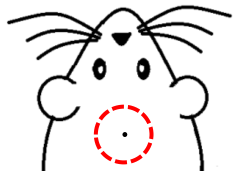	3	0(0.07 g)	--	--	--
Bourane et al, 2015	Nape Randomly	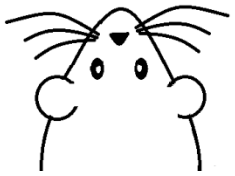	5	~10%(0.07 g) ~10%(0.16 g)	400 ng in NPY Abl mice	~50	Normal
Feng et al, 2018	Nape	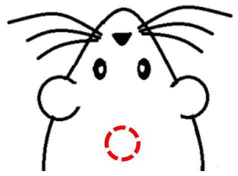	3 per site 5 sites	~16%(0.07 g) ~5% (0.16 g)	--	--	--
Pan et al, 2019	Ear	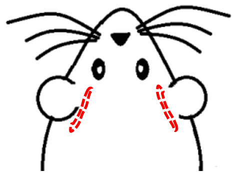	5	~80%(0.07 g) ~75%(0.16 g)	400 ng in naïve mice	~50	Normal
Acton et al, 2019	Nape Randomly	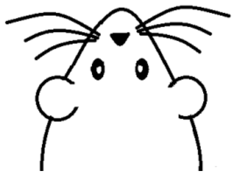	10	20%~40% (0.16 g)	400 ng in naïve mice	~100	Normal
Chen et al, 2020*	Nape	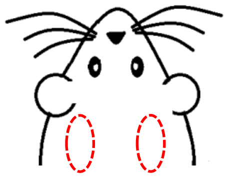	10	~40%(0.07 g) ~30%(0.16 g)	400 ng in naïve mice	~20	Normal
					500 ng in naïve mice	~5	Lost

* Present manuscript

Supplementary Table 2. Comparison of *Ucn3*^{tdTom} and *Tac2*^{tdTom} neurons.

	**Ucn 3**	**Tac 2**
Distribution	Lamina II-III	Exclusively Lamina IIi-IIIo
Aβ input	Yes	Yes
Feed-forward inhibition	Yes	Yes
Sensitization of Aβ evoked AP in dry skin itch model	Yes	Yes
Firing pattern in dry skin itch model	Unchanged	Unchanged
