# Supplementary material for: A spinal neural circuitry for converting touch to itch sensation
Source: Nat Commun. 2020 Oct 8;11:5074. doi: 10.1038/s41467-020-18895-7 (PMC7545208; doi:10.1038/s41467-020-18895-7)
Supplement: Supplementary file 2 — Reporting Summary [file 41467_2020_18895_MOESM2_ESM.pdf]

nature research

Corresponding author(s): Zhou-Feng Chen
Last updated by author(s): Aug 25, 2020

Reporting Summary

Nature Research wishes to improve the reproducibility of the work that we publish. This form provides structure for consistency and transparency in reporting. For further information on Nature Research policies, see Authors & Referees and the Editorial Policy Checklist .

Statistics

For all statistical analyses, confirm that the following items are present in the figure legend, table legend, main text, or Methods section.

n/a	Confirmed	
☐	☒	The exact sample size (n) for each experimental group/condition, given as a discrete number and unit of measurement
☐	☒	A statement on whether measurements were taken from distinct samples or whether the same sample was measured repeatedly
☐	☒	The statistical test(s) used AND whether they are one- or two-sided<br>*Only common tests should be described solely by name; describe more complex techniques in the Methods section.*
☐	☒	A description of all covariates tested
☐	☒	A description of any assumptions or corrections, such as tests of normality and adjustment for multiple comparisons
☐	☒	A full description of the statistical parameters including central tendency (e.g. means) or other basic estimates (e.g. regression coefficient) AND variation (e.g. standard deviation) or associated estimates of uncertainty (e.g. confidence intervals)
☐	☒	For null hypothesis testing, the test statistic (e.g. F, t, r) with confidence intervals, effect sizes, degrees of freedom and P value noted<br>*Give P values as exact values whenever suitable.*
☒	☐	For Bayesian analysis, information on the choice of priors and Markov chain Monte Carlo settings
☒	☐	For hierarchical and complex designs, identification of the appropriate level for tests and full reporting of outcomes
☒	☐	Estimates of effect sizes (e.g. Cohen's d, Pearson's r), indicating how they were calculated

Our web collection on statistics for biologists contains articles on many of the points above.

Software and code

Policy information about availability of computer code

Data collection	NIS Elements Ar, version 4.10 (Nikon), pClamp version 10.7 (Molecular Devices)
Data analysis	Prism, version 7.0d (GraphPad), Clampfit 10.7, Minianalysis 6.0.1 (Synaptosoft), Origin 2015 (OriginLab)

For manuscripts utilizing custom algorithms or software that are central to the research but not yet described in published literature, software must be made available to editors/reviewers. We strongly encourage code deposition in a community repository (e.g. GitHub). See the Nature Research guidelines for submitting code & software for further information.

Data

Policy information about availability of data

All manuscripts must include a data availability statement. This statement should provide the following information, where applicable:

- Accession codes, unique identifiers, or web links for publicly available datasets
- A list of figures that have associated raw data
- A description of any restrictions on data availability

The authors declare that all data supporting the findings of this study are available within the paper and its supplementary information files. Source data are provided with this paper.

Field-specific reporting

Please select the one below that is the best fit for your research. If you are not sure, read the appropriate sections before making your selection.

☒ Life sciences ☐ Behavioural & social sciences ☐ Ecological, evolutionary & environmental sciences

October 2018

Life sciences study design

All studies must disclose on these points even when the disclosure is negative.

Sample size	Sample size were based on previous experience (Sun and Chen, 2007, Sun et. al., 2009) and an online Power/Sample Size Calculator at https://www.stat.ubc.ca/~rollin/stats/ssize/n2.html.
Data exclusions	There were no missing data. Data outlier was not found by the definition as a data point that is located outside 1.5 times the interquartile range above the upper quartile and below the lower quartile.
Replication	All the experiments were repeated successfully at least two times to make sure the results are repeatable.
Randomization	Animals were randomly assigned to experimental groups.
Blinding	Experimenters were blinded to group allocation during data collection and data analysis.

Reporting for specific materials, systems and methods

We require information from authors about some types of materials, experimental systems and methods used in many studies. Here, indicate whether each material, system or method listed is relevant to your study. If you are not sure if a list item applies to your research, read the appropriate section before selecting a response.

Materials & experimental systems

n/a	Involved in the study	
☐	☒	Antibodies
☒	☐	Eukaryotic cell lines
☒	☐	Palaeontology
☐	☒	Animals and other organisms
☒	☐	Human research participants
☒	☐	Clinical data

Methods

n/a	Involved in the study	
☒	☐	ChIP-seq
☒	☐	Flow cytometry
☒	☐	MRI-based neuroimaging

Antibodies

Antibodies used	chicken anti-GFP (Aves Labs, GFP-1020), rabbit anti-c-Fos (Abcam, ab190289), rabbit anti-lmx1b (Invitrogen, Cat.PA5-34471), rabbit anti-Pax2 (Invitrogen, Cat.71-6000) and rabbit anti-NKB (Novus, Cat.NB300-201). Alexa-Fluor 488 conjugated donkey anti-chicken (Jackson ImmunoResearch Laboratories, 703-545-155), Cy3-conjugated donkey anti-rabbit (Jackson ImmunoResearch Laboratories, 711-165-152), 488- conjugated donkey anti-rabbit (Jackson ImmunoResearch Laboratories, 711-545-152) .
Validation	1. Chicken anti-GFP antibody was validated by Zylka et al., 2005 and the manufacture. "Antibodies were analyzed by western blot analysis (1:5000 dilution) and immunohistochemistry (1:500 dilution) using transgenic mice expressing the GFP gene product. Western blots were performed using BlokHen® (Aves Labs) as the blocking reagent, and HRP-labeled goat anti-chicken antibodies (Aves Labs, Cat. #H-1004) as the detection reagent. Immunohistochemistry used tetramethyl rhodamine-labeled anti-chicken IgY." https://www.aveslabs.com/products/green-fluorescent-protein-gfp-antibody?_pos=1&_sid=34576fa2a&_ss=r<br>2. Rabbit anti-c-Fos antibody was validated for immunohistochemistry on mouse brain by the manufacture.<br>3. Rabbit anti-lmx1b antibody was validated for immunohistochemistry on mouse tissues by the manufacture<br>4. Rabbit anti-Pax2 antibody was validated for immunohistochemistry on mouse spinal cord tissues by Jing Huang et al. (Nature Neuroscience, 2018) and the manufacture.<br>5. rabbit anti-NKB antibody was validated for immunohistochemistry on mouse brain tissues by Fergani C et al. (Elife, 2018) and True C et al. (Endocrinology, 2015).

Animals and other organisms

Policy information about studies involving animals; ARRIVE guidelines recommended for reporting animal research

Laboratory animals	Adult male mice between 7 and 12 weeks old were used for experiments. The mouse lines are C57BL/6J (Stock no.000664, Jax mice), Tac2Cre mice, Grpr-iCre mice, Ai32 mice (Stock no. 024109, Jax mice), Ai9 mice (Stock no.007909, Jax mice), Lbx1-flpo mice, and Tau-DTR mice.
Wild animals	The study did not involve wild animals
Field-collected samples	The study did not involve samples collected from field.

Ethics oversight	All animal studies conformed to guidelines set by the National Institutes of Health and the International Association for the Study of Pain and were reviewed and approved by the Institutional Animal Care and Use Committee (IACUC) at Washington University School of Medicine.

Note that full information on the approval of the study protocol must also be provided in the manuscript.
