# Supplementary material for: A spinal neural circuitry for converting touch to itch sensation
Source: Nat Commun. 2020 Oct 8;11:5074. doi: 10.1038/s41467-020-18895-7 (PMC7545208; doi:10.1038/s41467-020-18895-7)
Supplement: Supplementary file 3 — Description of Additional Supplementary Files [file 41467_2020_18895_MOESM3_ESM.pdf]

**Description of Additional Supplementary Files**

File Name: Supplementary Movie 1
Description: Scratching behavior between chemogenetic inhibition of Tac2 neurons and Tac2WT mice in response to mechanical stimulation (0.07 g von Frey hair force). 10 stimuli were delivered to the nape area at an interval of 10 s.

File Name: Supplementary Movie 2
Description: Scratching behavior between control and BB-sap 500 ng treated mice in response to mechanical stimulation (0.07 g von Frey hair force). 10 stimuli were delivered to the nape area at an interval of 10 s.
